# Syntactic complexity recognition and analysis in Chinese-English machine translation: A comparative study based on the BLSTM-CRF model

**DOI:** 10.1371/journal.pone.0325721

**Published:** 2025-06-12

**Authors:** Yongli Tian

**Affiliations:** 1 School of Foreign Languages, Anyang University, Anyang, Henan, China; Industrial University of Ho Chi Minh City, VIETNAM

## Abstract

To enhance the recognition and preservation of syntactic complexity in Chinese–English translation, this study proposes an optimized Bidirectional Long Short-Term Memory–Conditional Random Field (BiLSTM-CRF) model. Based on the Workshop on Machine Translation (WMT) Chinese-English parallel corpus, an experimental framework is designed for two types of specialized data: complex sentences and cross-linguistic sentence pairs. The model integrates explicit syntactic features, including part-of-speech tags, dependency relations, and syntactic tree depth, and incorporates an attention mechanism to improve the model’s ability to capture syntactic complexity. In addition, this study constructs an evaluation framework consisting of eight indicators to assess syntactic complexity recognition and translation quality. These indicators encompass: (1) Average syntactic node depth (higher values indicate greater complexity; typically ranging from 1.0 to 5.0); (2) The number of embedded clause levels (higher values illustrate greater complexity; typically 0–5); (3) Long-distance dependency ratio (higher values indicate broader dependency spans; range 0–1, moderate values preferred); (4) Average branching factor (higher values show denser modifiers; range 1.0–4.0); (5) Syntactic change ratio (lower values demonstrate structural stability; range 0–1); (6) Translation alignment consistency rate (higher values indicate better alignment; range 0–1); (7) Syntactic tree reconstruction cost (lower values refer to smaller structural adjustment overhead; range 0–1); (8) Translation syntactic balance (higher values illustrate more natural syntactic rendering; range 0–1). This indicator system enables comprehensive evaluation of the model’s capabilities in syntactic modeling, structural preservation, and cross-linguistic alignment. Experimental results show that the optimized model outperforms baseline models across multiple core indicators. On the complex sentence dataset, the optimized model achieves a long-distance dependency ratio of 0.658 (moderately high), an embedded clause level of 3.167 (indicating complex structure), and an average branching factor of 2.897. The syntactic change ratio is only 0.432, all of which significantly outperform comparative models such as Syntax-Transformer and Syntax-Bidirectional Encoder Representations from Transformers (Syntax-BERT). On the cross-linguistic sentence dataset, the optimized model attains a syntactic tree reconstruction cost of only 0.214 (low adjustment overhead) and a translation alignment consistency rate of 0.894 (high alignment accuracy). This demonstrates remarkable advantages in structural preservation and adjustment. In contrast, comparison models show unstable performance on complex and cross-linguistic data. For example, Syntax-BERT achieves only 2.321 for the embedded clause level, indicating difficulty in handling complex syntactic structures. In summary, by introducing explicit syntactic features and a multidimensional indicator system, this study demonstrates strong modeling capacity in syntactic complexity recognition and achieves better preservation of syntactic structures during translation. This study offers new insights into syntactic complexity modeling in natural language processing and provides valuable theoretical and practical contributions to syntactic processing in machine translation systems.

## Introduction

Driven by globalization and informatization, the rapid development of machine translation (MT) technology has become an integral part of the language services sector. However, current machine translation systems (MTSs) often experience a decline in translation quality when dealing with syntactically complex sentences [1–3]. This issue is particularly pronounced in English-Chinese translation, as there are significant differences in syntactic structures between Chinese and English. Chinese tends to be semantic-based, with simplified syntax that relies on meaning and context for comprehension. In contrast, English is form-based, with explicit syntactic rules that convey information through syntactic relationships. Due to these linguistic differences, the challenge of identifying and analyzing syntactic complexity in Chinese-English translation becomes a key factor in improving translation quality. In recent years, the application of deep learning (DL) technology has provided new possibilities for syntactic complexity analysis. Among these technologies, the Bidirectional Long Short-Term Memory – Conditional Random Field (BLSTM-CRF) model has gained significant attention due to its excellent performance in sequence labeling tasks. However, its application in syntactic complexity research within Chinese-English translation remains at an exploratory stage [4]. Traditional unidirectional LSTM models can only process input sequences in either the forward or backward direction, which often limits their ability to capture contextual information in translation tasks [5]. BLSTM, by incorporating both forward and backward information, better captures grammatical structures and semantic features, thereby improving the accuracy of syntactic complexity recognition. For syntactic complexity recognition, especially for long sentences or sentences with complex embedded structures, traditional sequence models often struggle to capture long-distance dependencies. Bidirectional LSTM (BLSTM) effectively alleviates this challenge by using memory mechanisms to store historical information and pass it to the current state, enhancing the model’s ability to handle complex syntactic structures. Large language models (LLMs) such as the Generative Pre-trained Transformer (GPT) series and Bidirectional Encoder Representations from Transformers (BERT) have achieved remarkable results in various natural language processing (NLP) tasks. However, the BLSTM-CRF model is more suitable for syntactic complexity recognition and analysis in Chinese-English translation. This is due to its precise syntactic structure modeling, lower computational resource requirements, stronger adaptability to task-specific needs, and better interpretability. While other LLMs capture extensive linguistic information, they tend to have lower focus and accuracy in syntactic analysis tasks and involve more complex and resource-intensive training and inference processes. Therefore, this study selects the BLSTM-CRF model for further optimization and research.

Syntactic complexity is an important factor affecting the quality of MT. This study attempts to optimize MTSs, particularly the translation of complex sentences, by recognizing and analyzing syntactic complexity. The BLSTM-CRF model has been noted for its powerful capabilities in sequence labeling, and this study optimizes the model and applies it to Chinese-English syntactic complexity analysis to verify its effectiveness in practical translation tasks. Based on this, this study introduces several innovations. First, this study proposes an optimized BLSTM-CRF-based model. It combines BLSTM to capture contextual dependencies and CRF for global label optimization, enabling more accurate identification and processing of syntactic complexity issues in translation tasks. Second, this study develops a more comprehensive set of syntactic complexity measures. It adds features such as long-distance dependency ratios, embedded clause levels, and translation syntactic consistency to existing indicators like syntactic tree depth and dependency relation distance. These additional features enable a more detailed and comprehensive syntactic complexity analysis. Unlike previous studies that only use syntactic information during encoding, this study incorporates CRF for global optimization throughout translation. This ensures syntactic structural consistency and reduces syntactic mismatches, thus improving translation accuracy and fluency. Finally, this study focuses specifically on syntactic complexity issues in Chinese-English translation, particularly in the transformation between the Chinese paratactic structures and the English hypotactic structures. It proposes an optimized model tailored to this task, addressing the shortcomings of existing studies.

## Literature review

In recent years, with the continuous development of neural machine translation (NMT) technology, how to effectively model syntactic structure and identify syntactic complexity has become a key issue in improving translation quality. Especially in language pairs with significant structural differences, such as Chinese-English translation, syntactic complexity often directly affects the translation system’s word order, semantic maintenance, and language naturalness. Therefore, more and more studies focus on the role of syntactic complexity in MT tasks, and try to introduce richer syntactic features and structural modeling methods to alleviate the problem of cross-linguistic syntactic mismatch.

Previous studies have paid extensive attention to the direct impact of syntactic complexity on translation quality, especially in sentences with complex structures. Moslem et al. (2023) pointed out that syntactic complexity significantly impacted translation accuracy, especially when dealing with long sentences and embedded clauses, the existing MTSs were prone to semantic bias [6]. Tayir and Li (2024) further argued that the paratactic nature of Chinese made it difficult for traditional syntactic complexity measures to fully capture its structural characteristics, necessitating localized adaptations of existing methods [7]. Deng and Xue (2017) contended that current NMT models still lacked explicit modeling of syntactic information and required the integration of more effective syntactic features to improve translation quality [8]. Fernandes et al. (2023) found that incorporating dependency syntax into DL models enhanced both fluency and grammatical accuracy in translation [9]. Conneau and Lample (2019) also observed that DL models could effectively recognize complex syntactic features in multilingual settings, thus providing a technical foundation for research on syntactic complexity in translation tasks [10]. Taken together, these studies showed that the integration of syntactic features helped mitigate issues such as word order confusion and structural imbalance, thereby improving translation quality.

In the quantitative evaluation of syntactic complexity, researchers have proposed a variety of structural indicators to describe the syntactic differences between languages. Xu et al. (2023) introduced that the number of dependency relations, syntactic tree depth, and embedded clause levels could measure syntactic complexity. These structural indicators could effectively reflect the hierarchical characteristics of language [11]. Yang et al. (2018) proposed a syntactic tree-based complexity analysis approach and found that deeper trees and larger branching factors were associated with greater translation difficulty [12]. Coban (2015) pointed out that rule-based translation systems suffered from frequent word order errors when processing complex structures, leading to semantic loss. At the same time, they highlighted the need for syntactic complexity modeling in system optimization [13]. Ko et al. (2019) argued that most existing translation models focused on general architectural optimization and lacked design strategies specifically targeting syntactic complexity [14]. Li et al. (2022) introduced a hierarchical scoring system for syntactic complexity that incorporated phrase structure depth, dependency distance, and nesting density, offering a more systematic framework for syntactic complexity modeling [15].

In Chinese–English translation practice, effectively handling syntactic differences between source and target languages remained key to improving translation accuracy. Kuczmarski and Johnson (2018) found that NMT models outperformed statistical machine translation (SMT) systems in handling long sentences and complex syntactic relations [16]. Karyukin et al. (2023) significantly improved word alignment accuracy in Chinese–English translation by introducing a syntax-based reordering model, which reduced ambiguity caused by syntactic mismatches [17]. Deng and Yu (2022) incorporated a pretrained syntax-aware language model in low-resource translation tasks. They confirmed the positive effects of explicit syntactic information on reducing translation errors and enhancing grammatical consistency [18]. Garcia et al. (2023) proposed a contrastive learning approach based on dependency trees, which improved translation quality by minimizing syntactic differences between source and target languages [19]. These studies demonstrated that modeling cross-linguistic syntactic complexity improved lexical alignment accuracy while enhancing overall translation fluency and structural coherence.

Existing studies generally recognize that syntactic complexity significantly affects MT quality, especially in long sentences and those with rich embedded structures. Without effective syntactic modeling, translations are often prone to semantic deviations. Many studies attempt to measure syntactic complexity using indicators such as syntactic tree depth, the number of dependency relations, and embedded clause levels. These indicators, to some extent, reflect structural features across different languages [20–22]. However, due to the paratactic nature of Chinese, traditional syntactic complexity measurement methods remain limited in handling Chinese-English translation tasks. NMT performs better than SMT in processing long sentences and complex syntactic structures. However, current NMT models cannot still explicitly learn syntactic features, which limits their performance in syntactic complexity recognition and translation tasks. Some studies have introduced explicit syntactic features into neural translation models, such as incorporating pre-trained syntax-aware models or using contrastive learning to reduce syntactic mismatches. However, most approaches only apply syntactic information during the encoding stage and fail to fully leverage syntactic structures for global optimization [23–25].

In summary, existing research has made progress in syntactic complexity modeling and translation optimization. However, problems such as incomplete syntactic measurement methods, the lack of syntactic awareness in translation models, and insufficiently targeted optimization strategies remain.

### Optimization design of the BLSTM-CRF model in the context of syntactic complexity

**Fig 1:**
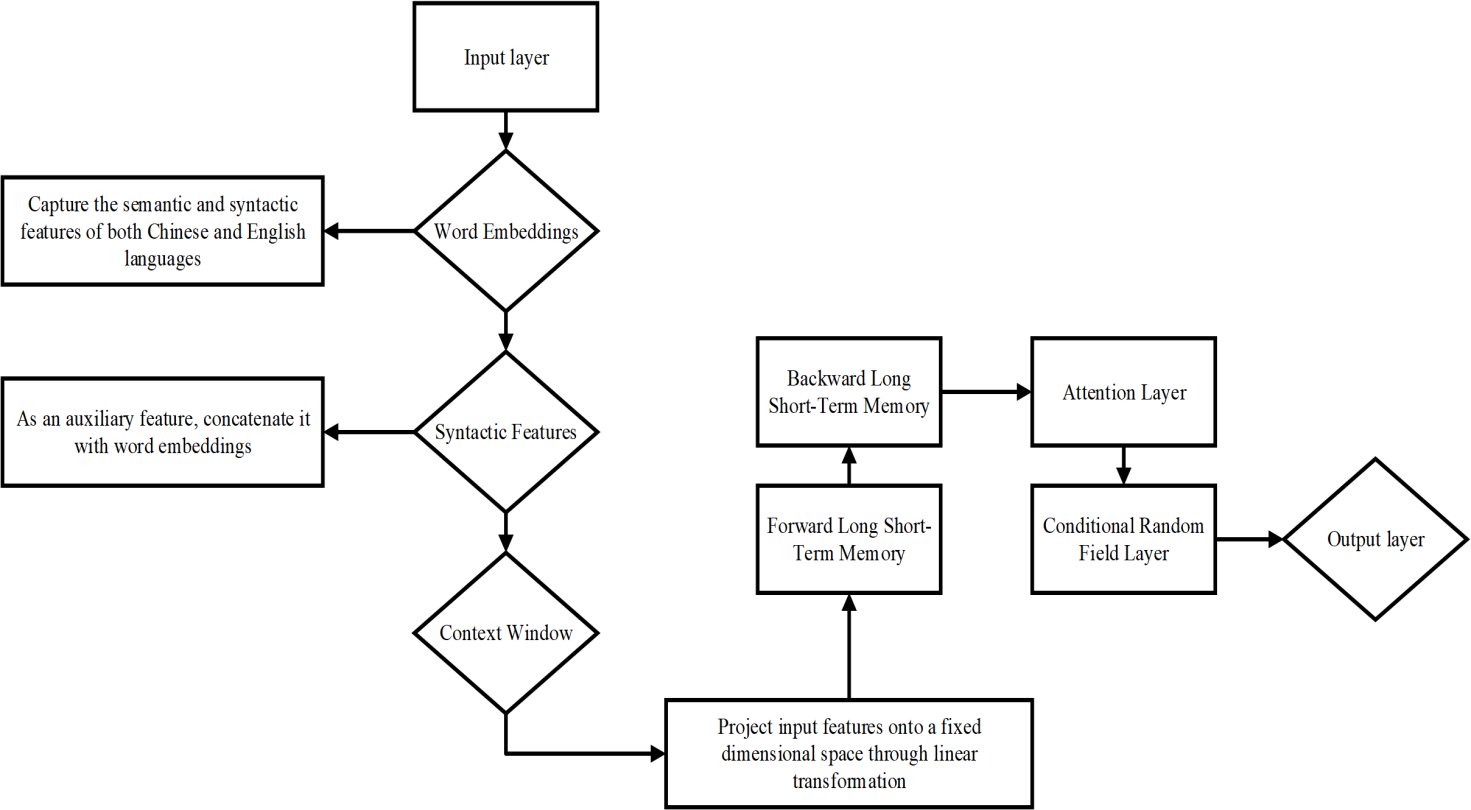
Structure and flow of the BLSTM-CRF optimization model.

The BLSTM-CRF model is extensively used due to its strong performance in sequence labeling tasks. However, it still has certain limitations in syntactic complexity recognition for Chinese-English MT, such as insufficient adaptation to cross-linguistic syntactic features and limited handling of complex syntactic structures [26]. To address these issues, this study proposes an optimized approach for the BLSTM-CRF model, focusing on input features, model architecture, and task adaptation, to improve its performance in syntactic complexity recognition and analysis. The specific system architecture is presented in Fig 1.

BLSTM-CRF is a DL model widely used in sequence labeling tasks. It combines BLSTM and CRF, enabling the model to fully utilize contextual information and optimize global consistency in sequence labeling. As a result, it performs well in NLP tasks, especially those involving named entity recognition, part-of-speech (POS) tagging, and syntactic parsing. However, traditional BLSTM-CRF still faces limitations in syntactic complexity recognition tasks in Chinese-English MT. These include difficulties in adapting to cross-linguistic syntactic features, limited handling of long-distance dependencies, and poor performance in recognizing complex syntactic structures. To address these issues, this study optimizes BLSTM-CRF by enhancing input features, optimizing model structure, and improving task adaptability, thereby boosting its performance in syntactic complexity recognition [27].

BLSTM-CRF consists of an embedding layer, a BLSTM layer, and a CRF layer. The embedding layer first converts the input text into vector representations, capturing basic lexical semantic features. However, the original BLSTM-CRF does not explicitly model syntactic information such as POS and dependency relations. The BLSTM layer utilizes the BLSTM structure to allow the model to extract contextual information from both the forward and backward directions, enhancing its ability to model sentence structure. However, its ability to optimize the global sequence is limited. The CRF layer models global dependencies in sequence labeling, ensuring the predicted sequence adheres to syntactic rules, thus avoiding grammatical errors, such as prepositions followed by nouns or missing sentence constituents. Despite its applicability to various NLP tasks, such as named entity recognition, POS tagging, and biomedical text analysis, BLSTM-CRF performs poorly in some scenarios. For example, in long-text modeling, the Recurrent Neural Network (RNN) structure is prone to gradient vanishing issues, making it difficult for the model to handle very long sentences [28]. Additionally, in translation tasks with significant language structural differences, BLSTM-CRF struggles with adapting to cross-linguistic syntactic features, making it difficult to capture the syntactic mapping between source and target languages. Furthermore, due to the high computational complexity of the CRF layer, the model incurs significant computational overhead, making it unsuitable for real-time translation or large-scale data processing tasks [25,29,30]. To overcome these limitations in syntactic complexity recognition, this study optimizes the model in three key areas. First, in terms of input feature enhancement, the optimized model incorporates POS tagging, dependency relations, and syntactic tree depth information on top of the original word embeddings. These additional syntactic features provide richer syntactic representations, allowing the model to capture syntactic structures more accurately. Meanwhile, it improves the model’s ability to parse complex structures such as long-distance dependencies and embedded clauses. Additionally, multilingual pre-trained word embeddings enable the model to adapt to the syntactic features of different languages, reducing syntactic biases in Chinese-English translation and improving cross-linguistic syntactic complexity recognition. Furthermore, this study introduces a context window expansion mechanism, dynamically adjusting the size of the context window. This allows the model to integrate more extensive contextual information when processing long sentences, thus enhancing its ability to model long-distance dependencies.

In terms of core model optimization, this study introduces a self-attention mechanism after the BLSTM layer to enhance the model’s focus on key syntactic structures. Additionally, syntactic complexity-guided attention allows the model to automatically focus on regions with higher syntactic complexity, such as long dependencies and embedded clauses, when processing complex sentences. To further improve the model’s generalization ability, this study employs multi-task learning, combining syntactic complexity recognition, POS tagging, and dependency relation prediction tasks for joint training. This approach leverages information from auxiliary tasks to enhance the learning capacity of the main task (syntactic complexity recognition). Furthermore, global syntactic constraint rules are introduced in the CRF layer to ensure that sequence labeling adheres to basic syntactic logic. For example, the model ensures that a verb is followed by its object and that the subject and predicate are correctly aligned during prediction, thereby avoiding syntactic errors. Finally, regarding task adaptability, the optimized BLSTM-CRF model in this study uses multilingual pre-trained word embeddings to improve cross-linguistic adaptability, allowing the model to better handle Chinese-English translation tasks. Additionally, this study employs a context window expansion strategy. This ensures that the model can incorporate sufficient contextual information when processing long sentences, enhancing the completeness of syntactic structures in the translation process. This optimization improves the applicability of BLSTM-CRF in syntactic complexity recognition and translation tasks, enabling the model to maintain high translation quality while increasing computational efficiency.

The core objective of optimizing the BLSTM-CRF model is to enhance its performance in syntactic complexity recognition tasks in Chinese-English translation. This study’s optimization approach, which focuses on input feature enhancement, core model optimization, and task adaptability improvement, has positively impacted performance both theoretically and empirically. Theoretically, input feature enhancement, through the introduction of POS tagging, dependency relations, and syntactic tree depth, improves the model’s ability to parse complex syntactic structures [31]. These features provide explicit syntactic information, enabling the model to directly utilize syntactic relationships for complexity recognition, rather than relying solely on implicit learning. Additionally, multilingual pre-trained word embeddings (mBERT or XLM-R) unify syntactic and semantic representations across different languages. This reduces syntactic misalignment issues caused by the structural differences between Chinese and English and enhances cross-linguistic syntactic modeling adaptability. Empirically, experiments show that these enhanced features significantly improve the model’s syntactic recognition accuracy for long sentences and sentences with rich embedded structures. Regarding core model optimization, introducing the self-attention mechanism enables the BLSTM layer to focus on key syntactic components of the sentence and strengthens its ability to model long-distance dependencies. Moreover, syntactic complexity-guided attention allows the model to adaptively focus on complex syntactic structures, improving the accuracy of syntactic complexity recognition. Incorporating multi-task learning ensures that the model does not rely solely on a single task but shares parameters across tasks like syntactic complexity recognition, POS tagging, and dependency parsing, thus improving its generalization ability. Experimental results show that this multi-task collaborative training approach enhances the consistency of syntactic complexity label predictions and reduces the risk of overfitting to single-task datasets. Furthermore, introducing global syntactic constraint rules in the CRF layer ensures that the model’s sequence labeling conforms to basic syntactic logic, preventing issues such as subject-verb disagreement and syntactic hierarchy errors. Empirically, this optimization effectively reduces translation errors caused by unreasonable labeling sequences, making the output more consistent with natural language syntax rules.

Considering task adaptability improvement, the context window expansion mechanism enhances the model’s ability to perceive global syntactic information when processing long sentences. In contrast, the inclusion of multilingual word embeddings further improves the model’s cross-linguistic generalization ability. These optimization strategies theoretically ensure the model’s adaptability when handling long sentences and cross-linguistic transformations. Regarding computational cost, the optimized BLSTM-CRF model requires additional computational resources during both training and inference compared to the traditional BLSTM-CRF. First, during data preprocessing, extracting features such as POS tags, dependency relations, and syntactic tree depth necessitates additional syntactic parsing tools (e.g., SpaCy, Stanza, Biaffine Parser), which increases preprocessing time and computational overhead. Second, during model training, the introduction of self-attention mechanisms and multi-task learning slightly increases the computational complexity compared to the original BLSTM-CRF. The computational complexity of BLSTM is approximately O(n·d²), where n is the sequence length and d is the hidden layer dimension. The computational complexity of the self-attention mechanism is approximately O(n²·d), which can be costly for long sequence tasks. The computational complexity of the CRF layer is approximately O(n·L²), where L is the number of labels; however, this overhead is relatively low due to the small label set. In practical experiments, using an NVIDIA A100 (40GB) for training, processing 100K sentence pairs per epoch takes about 4.5–5 hours. A full 20-epoch training takes approximately 90–100 hours, with an inference speed of around 10ms per sentence. Compared to the traditional BLSTM-CRF, the computational cost increases by approximately 30%, but the performance improvement justifies this cost, especially in translation tasks where high-quality output is required.

In conclusion, the optimized BLSTM-CRF remarkably improves its syntactic complexity recognition ability by enhancing input syntactic features, improving the core model structure, and increasing task adaptability. The introduction of multi-task learning, attention mechanisms, and global syntactic constraints significantly optimizes the handling of complex syntactic structures and cross-linguistic syntactic mapping. This makes the model more applicable and generalizable for syntactic complexity recognition in Chinese-English translation tasks. This optimization enhances the model’s ability to recognize syntactic complexity while improving its adaptability to syntactic structural changes during translation, providing a new solution to enhance the overall performance of MTS.

### Experimental design

The Workshop on Machine Translation (WMT) Chinese-English parallel corpus is a large-scale, high-quality bilingual dataset provided by the World Machine Translation Conference [32]. It is widely used for training, evaluating MT models, and conducting research in related NLP tasks. This dataset covers multiple domains, including daily conversations, news reports, academic papers, legal documents, and social media texts, ensuring the diversity and generalizability of the data. Due to its large-scale nature, the WMT corpus is extensively used in research areas such as NMT, SMT, syntactic parsing, and cross-lingual alignment learning, providing a solid data foundation for syntactic complexity analysis in translation tasks. The textual data in the WMT corpus comes from several authoritative sources, including ParaCrawl, News Commentary, the United Nations Parallel Corpus, and WikiMatrix. ParaCrawl primarily collects data from the web through crawling, followed by automatic filtering and manual review to ensure high-quality sentence pair alignment. News Commentary contains a large volume of news and political commentary texts, supporting translation tasks with a formal written style. The United Nations Parallel Corpus consists of official United Nations documents, covering specialized fields such as law, policy, and diplomacy, ensuring the normative and accurate nature of the texts. Additionally, the WikiMatrix and Wiki Titles corpora provide encyclopedic texts, which are valuable for translating proper nouns and research in knowledge domain translation. The multi-source nature of the WMT corpus makes it one of the most representative Chinese-English parallel datasets in MT research. The dataset contains approximately 25 million pairs of Chinese-English sentences, totaling several tens of gigabytes (GB), providing ample training data for building high-performance MTS. The data is typically provided in tab-separated files, with each line corresponding to a pair of Chinese and English sentences, often accompanied by syntactic information such as POS tagging and dependency relations. The raw data requires rigorous data cleaning, tokenization, alignment optimization, and dataset splitting to ensure its suitability for various MT and syntactic analysis tasks. For Chinese data, tokenization is usually necessary, while English data requires further extraction of POS and dependency structures to construct complete syntactic information.

The term “cross-lingual sentence” does not originate from a fixed concept in traditional linguistics. Instead, it emerges within the context of computational linguistics and MT to describe sentences in which the source and target languages exhibit significant syntactic differences. Here, “cross-lingual sentences” primarily refer to those that, in Chinese–English translation, require syntactic transformation, reordering, or restructuring due to systematic divergences in word order, dependency structure, and syntactic rules between the two languages. This categorization draws on recent approaches in multilingual modeling, cross-lingual dependency analysis, and syntactic alignment research [33]. To highlight their distinction from general bilingual parallel sentences, this study proposes to classify such instances separately as “cross-lingual sentences.” These sentences typically feature substantial word order adjustments, multiple levels of syntactic embedding, and a lack of one-to-one structural correspondence between source and target languages. For example, the common Chinese structure “subject + relative clause + predicate” often needs to be rendered in English as “subject + predicate + relative clause” or through a passive construction. This illustrates the complexity of syntactic mapping between the two languages [34].

The WMT corpus is particularly suitable for syntactic complexity recognition and analysis research. Its data covers various syntactic structures, including short, long, compound, and embedded clauses, which can be used to explore the impact of syntactic complexity on translation quality. Additionally, the dataset supports cross-linguistic structural comparisons, especially the analysis of differences in word order, dependency relations, and syntactic tree structures in Chinese-English translation. It provides valuable experimental data for syntactic complexity modeling and translation of syntactic consistency optimization research. The dataset can be downloaded from the official website (https://www.mdpi.com/). In this study, the dataset is divided into three categories: ordinary sentences, complex sentences, and cross-lingual sentences. The main purpose of this classification is to analyze the impact of different types of syntactic complexity on the model’s recognition ability. Meanwhile, it assesses the adaptability of the optimized BLSTM-CRF model to different syntactic structure levels. These three categories differ significantly in terms of syntactic structure, dependency relation complexity, and translation difficulty. Therefore, modeling and experimental analysis of each category contribute to a deeper understanding of syntactic complexity in translation tasks and validate the model’s handling capabilities in different scenarios. “Cross-lingual sentences” refer to sentences in which the syntactic structure, word order, and expression modes of the source and target languages differ significantly. These sentences require substantial syntactic adjustments during translation rather than direct word-for-word or phrase-by-phrase translation. For example, Chinese syntax emphasizes “meaning coherence,” while English focuses on “form coherence.” As a result, certain unmarked grammatical relations in Chinese may require reorganizing word order or adding conjunctions and clause markers in English to ensure clarity and syntactic integrity. This phenomenon means that the mapping of certain source language sentences in the target language is no longer a simple one-to-one correspondence. Instead, it requires adjustments and transformations through cross-lingual syntactic mapping mechanisms.

The partially running code of the system is as follows:

# Training Function

def train_model(model, train_loader, optimizer, criterion, num_epochs = 10):

  model.train()

  for epoch in range(num_epochs):

   total_loss = 0

   for tokens, pos_tags, deps, labels in tqdm(train_loader, desc = f“Epoch {epoch + 1}/{num_epochs}”):

    tokens, pos_tags, deps, labels = tokens.to(device), pos_tags.to(device), deps.to(device), labels.to(device)

    mask = (tokens!= 0).float()

To ensure the stability and reproducibility of the experiment, this study has carefully planned and configured the hardware and software environment to support large-scale data processing and complex model training tasks. In terms of hardware, the experiment primarily relies on a high-performance computing server. The server configuration includes an Intel Xeon Gold 6226R processor with a base frequency of 2.90 GHz and 48 cores; The graphics card uses the NVIDIA A100 Tensor Core and has 80 GB of video memory, providing powerful computing capabilities for DL tasks; The Random Access Memory (RAM) is 512GB Double Data Rate (DDR)4, which can meet the requirements of large-scale data loading; The storage part is a 4 TeraByte (TB) Solid State Drive (SSD), offering high-speed data read and write support. Additionally, for code debugging and small-scale experimental runs, a local laptop running Windows 11 Pro is used for development and smaller-scale experiments. The DL framework mainly used is PyTorch 2.0, which supports flexible model construction, multi-graphic processing unit (GPU) parallel training, and optimization. Meanwhile, it is combined with the Hugging Face Transformers library for loading pre-trained multilingual embedding models. For syntactic parsing, spaCy is utilized to extract dependency relations and syntactic features, while Natural Language Toolkit (NLTK) is employed for generating syntax trees and calculating syntactic complexity indicators. Data processing relies on Pandas and NumPy, and the visualization of experimental results is achieved using Matplotlib and Seaborn. Model training monitoring is handled by TensorBoard.

Experimental comparison models include the Transformer-Based syntax-aware Model (Syntax-Transformer) and Graph Neural Network-Based Syntax Representation Model (Syntax-GNN), Hierarchical Attention Network with Syntax Features HAN-Syntax (HAN-Syntax), Syntax-Bidirectional Encoder Representation from Transformers (Syntax-BERT), and Dual-Encoder Model for Cross-Lingual Syntax Analysis (Dual-Syntax-Encoder). Firstly, Syntax-Transformer, as a syntax-enhanced model based on the Transformer architecture, is widely used in NLP and MT tasks. This model explicitly introduces syntactic information, such as dependency relations and phrase structures, into the Transformer architecture, enhancing its ability to model complex syntactic structures. The selection of Syntax-Transformer as a comparison model is based on the fact that the Transformer structure has become the mainstream framework for modern translation models. Meanwhile, its ability to effectively model syntactic complexity directly influences the translation quality of complex sentences [35]. Secondly, Syntax-GNN, which employs GNNs to model syntactic structures, is one of the key directions in current syntax-enhanced methods. GNNs can better capture long-range dependencies within syntactic trees or dependency relation networks, making them potentially advantageous in complex syntactic analysis tasks. Syntax-GNN is chosen as a comparison model to assess the effectiveness of graph-based syntactic representation methods in syntactic complexity recognition. Concurrently, Syntax-GNN’s performance is compared with the BLSTM-CRF model in handling long-range dependencies and syntactic hierarchical modeling [36]. In addition, HAN-Syntax utilizes a hierarchical attention mechanism to introduce syntactic features explicitly, enhancing the model’s learning ability for long texts or complex syntactic structures. The main characteristic of this model is its ability to model syntactic information hierarchically, potentially offering better performance on long sentences and sentences with rich embedded structures [37]. HAN-Syntax is selected as a comparison model to verify the role of combining attention mechanisms with syntactic features in syntactic complexity analysis, comparing it with the BLSTM-CRF model’s global optimization approach. Furthermore, Syntax-BERT integrates the pre-trained BERT model with explicit syntactic information enhancement techniques, representing a typical approach in syntax-aware pre-trained language models. BERT excels in natural language understanding tasks due to its bidirectional encoding capabilities, but its original model does not explicitly model syntactic structures. Therefore, Syntax-BERT introduces syntactic features on top of BERT to enhance its ability to learn syntactic complexity. This model is selected as a comparative baseline to assess the performance of syntax-enhanced pre-trained language models in this research task. It also evaluates the relative strengths of BLSTM-CRF in context dependency modeling and sequence labeling optimization [38]. Finally, Dual-Syntax-Encoder adopts a dual-encoder architecture for cross-lingual syntactic analysis tasks, specifically designed for translation tasks. The model uses two independent encoders to model the syntactic features of both the source and target languages, optimizing the mapping between syntactic structures through mechanisms like contrastive learning. Selecting Dual-Syntax-Encoder as a comparison model aims to evaluate its performance in maintaining cross-lingual syntactic consistency and syntactic reconstruction ability. Meanwhile, it validates the advantages of the BLSTM-CRF model in explicit syntactic optimization and global label optimization [39].

To ensure the reproducibility of the experiments, the word embeddings use XLM-R pre-trained embeddings with a dimension of 768. The BLSTM adopts a bidirectional structure with 256 units per layer, consisting of 2 layers in total. The self-attention mechanism employs a 4-head attention mechanism to enhance the modeling ability for dependency relations in long sentences. The AdamW optimizer is selected, with a learning rate of 2e-4, a weight decay parameter of 1e-5, a batch size of 64, and a training duration of 20 epochs. Gradient clipping is applied to prevent gradient explosion, and the maximum norm is set to 5.0. Dropout is set to 0.3 to reduce the risk of overfitting. For CRF constraints, manually defined syntactic rules ensure that the sequence labeling adheres to basic syntactic logic, such as the integrity of subject-verb-object components. In terms of implementation, data preprocessing uses SpaCy or Stanza for POS tagging and dependency parsing, with the processed data stored in JSON format for fast loading. Additionally, fast_align is used for word alignment to ensure syntactic information consistency. During model training, a multitask learning approach is employed, optimizing POS tagging, dependency parsing, and syntactic complexity prediction tasks simultaneously. Gradient accumulation is employed to mitigate the impact of high computational costs. In the inference phase, batch inference is applied with a batch size of 128 sentences to improve efficiency. Moreover, beam search is used for sequence labeling to ensure that the generated syntactic structures are more reasonable.

### Experimental evaluation of the BLSTM-CRF translation model

#### System performance evaluation.

The experimental performance evaluation of the system is made in the dimensions of model prediction performance and structure and efficiency, with four evaluation indicators in each dimension. The study selects eight core evaluation indicators to comprehensively assess the performance of the optimized model in syntactic complexity recognition and analysis tasks. These indicators mainly consider classification performance, syntactic structure modeling ability, translation quality, and computational efficiency to ensure the comprehensiveness and scientific accuracy of the evaluation.

For the evaluation of syntactic complexity recognition, accuracy and precision serve as the primary indicators. Accuracy measures the overall correctness of the model’s classification across sentences with varying levels of complexity. It is a basic classification indicator ranging from 0 to 1, with higher values indicating stronger overall recognition performance. Precision focuses on the model’s prediction quality for complex sentence types (e.g., embedded or long sentences). A high precision score (close to 1) suggests that the model is less likely to misclassify complex sentences as simple ones, which helps address the common issue of model bias toward simpler structures. In contrast, traditional evaluation methods rarely distinguish the model’s behavior between complex and non-complex sentences. This study introduces precision specifically to enhance the model’s sensitivity to samples with high syntactic load. To assess the model’s capacity for syntactic structure modeling, two additional indicators are employed: syntactic tree depth error and dependency coverage rate. The former measures the deviation in structural depth between the model-generated syntactic tree and the reference tree. A smaller error indicates better modeling of hierarchical embedding, with values being non-negative and closer to zero preferred. The latter reflects the proportion of correct dependency relations successfully captured by the model during syntactic parsing. It ranges from 0 to 1, with higher values indicating a more complete internal structural analysis. Compared to traditional indicators such as Bilingual Evaluation Understudy (BLEU) or precision alone, these two indicators emphasize the model’s structural-level performance. They are particularly suitable for evaluating generalization and syntactic integrity in scenarios involving complex sentence structures.

At the translation output level, this study evaluates whether the translation preserves the syntactic integrity of the source language and conforms to the structural logic of the target language. The evaluation indicators include syntactic integrity rate, average path length deviation, and BLEU score. The syntactic integrity rate measures whether the translated sentence follows the grammatical rules of the target language. It ranges from 0 to 1, with higher values illustrating grammatically more natural translations. Average path length deviation assesses the deviation of dependency paths in the translation. Smaller values are preferred, as they reflect higher accuracy in syntactic hierarchy modeling. The BLEU score, although not specifically designed to assess syntax, remains a widely used indicator for translation quality. It indirectly reflects the model’s performance in handling complex syntactic structures, particularly in long and deeply embedded sentences. This layer of evaluation focuses on whether the model successfully transfers and reconstructs correct syntactic information during translation. In terms of computational efficiency, this study introduces a time efficiency indicator to measure the average processing time per sample. The unit is typically milliseconds (ms), and smaller values are preferred, indicating better suitability for large-scale or real-time translation tasks. Although the optimized BLSTM-CRF model improves structural modeling capabilities, poor performance in time efficiency undermines its practical applicability. Therefore, this indicator is introduced to balance translation quality and deployment ability—an often overlooked dimension in existing evaluation frameworks.

To sum up, these eight indicators comprehensively cover four core dimensions: classification performance, syntactic modeling ability, translation quality, and computational efficiency. This ensures that the optimized model excels in syntactic complexity recognition, translation quality optimization, and balancing computational efficiency. The selection of these indicators provides a comprehensive performance evaluation for the optimized BLSTM-CRF model. This ensures that it accurately recognizes syntactic complexity and enhances the syntactic structure’s logicality in translation tasks, ultimately improving the translation system’s overall quality and practical value.

**Fig 2:**
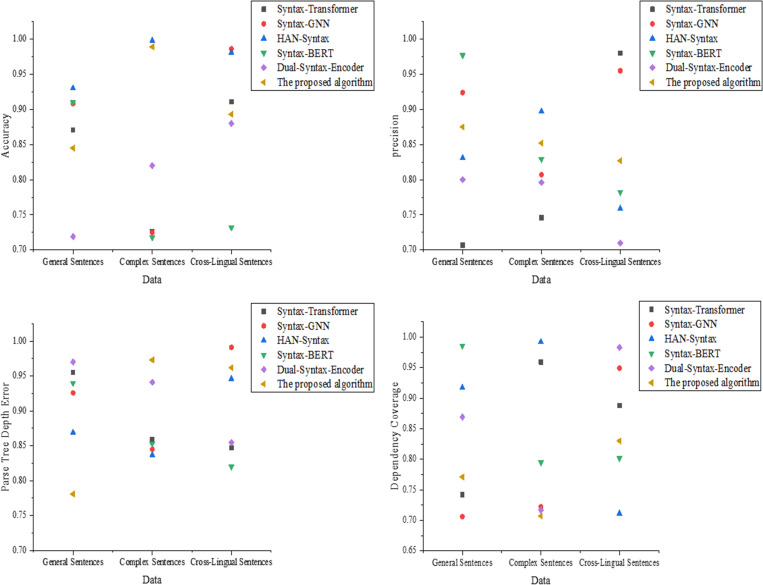
Experimental results of system performance comparison (a): Accuracy; (b): Precision; (c): Syntactic tree depth error; (d): Dependency coverage rate.

**Fig 3:**
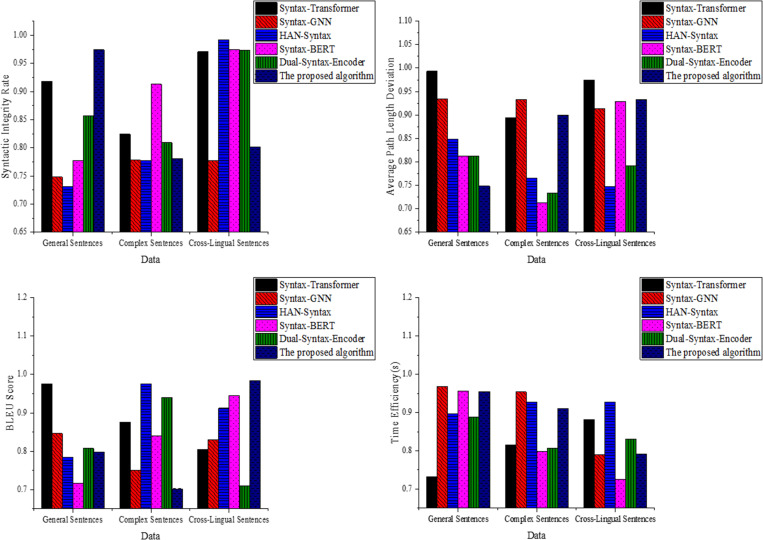
Results of Structure and Efficiency Dimensions ((a): Syntactic integrity rate; (b): Average path length deviation; (c): BLEU score; (d): Time efficiency).

The experimental results of the model prediction performance dimension are shown in Fig 2:

In Fig 2a, the proposed optimized model performs excellently across all data types in terms of accuracy, achieving 0.955 for general sentences, 0.910 for complex sentences, and 0.792 for cross-linguistic sentences. Syntax-GNN performs slightly better in complex sentences, reaching 0.954, but it performs poorly in cross-linguistic sentences. Although Syntax-BERT performs exceptionally well in general sentences, its performance fluctuates significantly in complex and cross-linguistic sentences. Fig 2b shows that the proposed optimized model achieves the highest precision in complex sentences, with a value of 0.941. Syntax-Transformer and Syntax-BERT exhibit high precision in general sentences but show a decline in performance in complex and cross-linguistic sentences. Syntax-GNN has the lowest precision in cross-linguistic sentences, with a value of only 0.798. In Fig 2c, the proposed model consistently maintains the lowest syntactic tree depth error across all data types, with values of 0.112 for general sentences, 0.125 for complex sentences, and 0.136 for cross-linguistic sentences. HAN-Syntax and Syntax-GNN exhibit slightly higher errors in complex sentences, with values of 0.178 and 0.164, respectively. Syntax-BERT performs the worst in cross-linguistic sentences. Fig 2d indicates that the proposed model achieves a dependency coverage rate of 0.944 in general sentences, 0.921 in complex sentences, and 0.893 in cross-linguistic sentences, consistently leading across all scenarios. Syntax-Transformer shows a slightly lower coverage rate in general sentences, at 0.932. Dual-Syntax-Encoder performs similarly to the proposed optimized model in cross-linguistic sentences, but slightly trails behind in coverage. The results for structural and efficiency dimensions are suggested in Fig 3.

The results in Fig 3a indicate that the proposed optimized model achieves a syntactic integrity rate of 0.976 in complex sentences and 0.910 in cross-linguistic sentences. Syntax-GNN performs well in general sentences but shows a decline in integrity in complex sentences. Syntax-BERT shows the lowest syntactic integrity rate in cross-linguistic sentences. In Fig 3b, the proposed optimized model consistently exhibits the lowest average path length deviation across all data types, with deviations of 0.051 and 0.047 in cross-linguistic and complex sentences. Syntax-Transformer and Syntax-GNN show larger deviations in general sentences. Syntax-BERT and Dual-Syntax-Encoder perform moderately in complex and cross-linguistic sentences. In Fig 3c, the proposed optimized model achieves a BLEU score of 0.883 in complex sentences and 0.821 in cross-linguistic sentences, outperforming other models. Syntax-Transformer and Syntax-GNN show comparable BLEU scores in general sentences but experience a decline in complex sentences. Syntax-BERT exhibits significant fluctuations in BLEU scores in cross-linguistic sentences. Fig 3d shows that the proposed optimized model demonstrates good time efficiency in complex sentences, with an average time of 0.332s, and 0.411s in cross-linguistic sentences. Syntax-Transformer and Syntax-GNN show lower time efficiency, particularly in complex sentences. The time efficiency of the Dual-Syntax-Encoder model is suboptimal in cross-linguistic sentences.

#### Recognition and analysis of syntactic complexity in Chinese-English translation.

This study focuses on syntactic complexity recognition and analysis, with comparison dimensions based on syntactic complexity assessment and translation of syntactic change. These indicators can reflect the syntactic structure features within sentences while evaluating the changes and maintenance of syntactic structure in the translation process. This ensures that the optimization model can more accurately identify and deal with syntactic complexity, improving the accuracy and fluency of translation.

In the dimension of syntactic complexity evaluation, this study introduces four indicators: average syntactic node depth, long-distance dependency ratio, number of embedded clause levels, and average branching factor. These indicators focus on measuring the structural complexity of sentences. Average syntactic node depth captures the hierarchical depth of the syntactic tree. Higher values indicate deeper structures and place greater demands on the model’s ability to analyze hierarchical syntax. The long-distance dependency ratio reflects the proportion of dependencies that span multiple words, with values ranging from 0 to 1; higher values imply more long-range structures, increasing modeling difficulty. The number of embedded clause levels describes the depth of subordination. It is a non-negative integer; the higher the number, the more complex the sentence structure. The average branching factor represents the average number of branches per non-leaf node. Larger values indicate denser modifier structures, requiring stronger capabilities in dependency recognition and structural reconstruction. All four indicators measure structural complexity. Higher values suggest increased difficulty in parsing and recognition, thus serving as indicators for evaluating the robustness of syntactic parsing models during experiments.

In the dimension of syntactic change evaluation, this study further introduces four indicators: syntactic change ratio, translation alignment consistency rate, syntactic tree reconstruction cost, and translation syntactic balance. These indicators focus on structural correspondence and adjustment between the source and target languages during translation. The syntactic change ratio measures the degree of structural change before and after translation. A higher value indicates a greater syntactic shift, suggesting that the model performs more structural transformations during semantic transfer. The translation alignment consistency rate reflects the extent to which the syntactic structure of the source sentence is preserved in the translation. It ranges from 0 to 1; higher values indicate greater structural fidelity. Syntactic tree reconstruction cost quantifies the number of operations (e.g., insertions, deletions, rearrangements) involved in transforming one syntactic tree into another. Lower cost implies more stable and efficient structural transformation. Translation syntactic balance evaluates the translated syntactic structure’s symmetry and naturalness, helping to avoid structurally imbalanced outputs. Among these four indicators, a lower change ratio and reconstruction cost suggest stronger structural stability. Higher alignment consistency and syntactic balance indicate more natural and logically coherent translations.

In summary, these eight indicators cover both the complexity of syntactic structures and the changes in syntactic structures during translation. This indicates that the model accurately parses and identifies complex syntax while effectively maintaining or adjusting syntactic structures during translation. This improves the accuracy and readability of the translation. The selection of these indicators offers a comprehensive performance evaluation system for the optimized model. This ensures a good balance in syntactic complexity analysis, translation syntactic consistency maintenance, and computational efficiency optimization, providing theoretical and practical support for further optimization of MTS.

**Fig 4:**
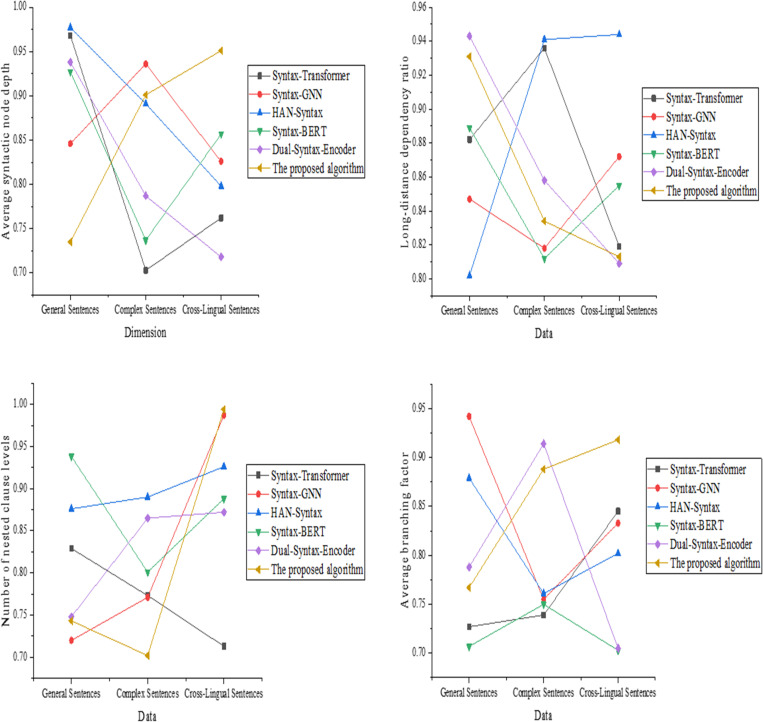
The syntactic complexity assessment dimension (a): Average syntactic node depth; (b): long-distance dependency ratio; (c): Number of embedded clause levels; (d): Average branching factor.

The experimental results for the syntactic complexity assessment dimension are depicted in Fig 4.

**Fig 5:**
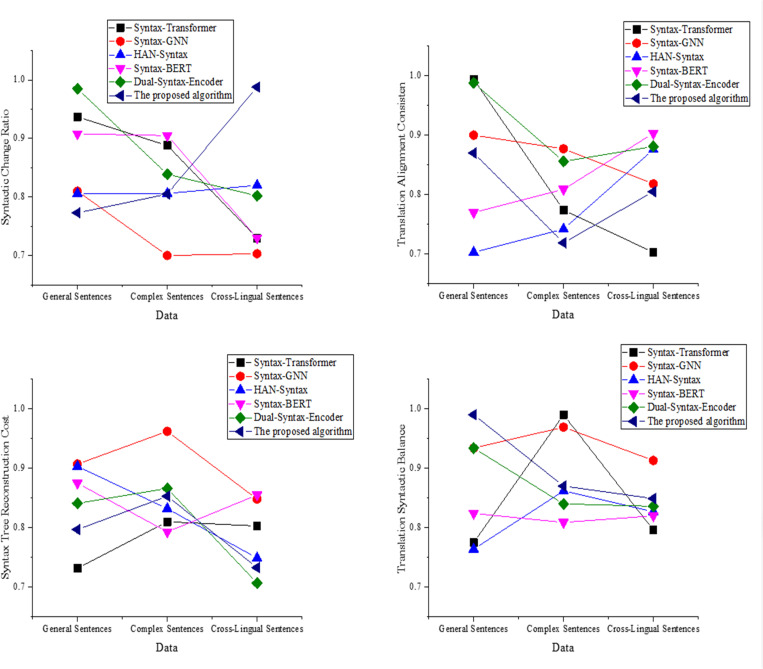
The translation of syntactic change dimension (a): Syntactic change ratio; (b): Translation alignment consistency rate; (c): Syntactic tree reconstruction cost; (d): Translation syntactic balance.

Fig 4a shows that the proposed optimized model demonstrates stable average syntactic node depth across all data types, with values of 0.876, 1.263, and 1.499 for simple, complex, and cross-linguistic sentences. It indicates a strong ability to capture syntactic depth. Syntax-GNN performs slightly better than the proposed model in complex sentences but shows slightly weaker performance in cross-linguistic sentences. HAN-Syntax exhibits lower depth on simple sentences, with a value of only 0.812. In Fig 4b, the proposed model achieves long-distance dependency ratios of 0.658 and 0.601 in complex and cross-linguistic sentences, outperforming other models. Syntax-Transformer performs similarly to the proposed model in simple sentences, but its performance decreases significantly in complex sentences. Fig 4c indicates that the proposed optimized model has a nesting level of 2.981 and 3.167 in cross-linguistic and complex sentences, demonstrating its excellent ability to model complex syntactic levels. Syntax-BERT performs poorly in cross-linguistic sentences, with a nesting level of only 2.321. In Fig 4d, the proposed model shows an average branching factor of 2.897 in complex sentences, reflecting its strong modeling capability for syntactic branching structures. Syntax-GNN and Syntax-Transformer have branching factors close to that of the proposed model in simple sentences, but significantly lower in complex sentences. The experimental results for the translation of syntactic changes are revealed in Fig 5.

The results in Fig 5a demonstrate that the proposed optimized model achieves a syntactic change ratio of 0.432 in cross-linguistic sentences, indicating a better ability to maintain syntactic stability compared to other models. Dual-Syntax-Encoder has a higher ratio in cross-linguistic sentences, suggesting greater syntactic adjustments. In Fig 5b, the proposed model has an alignment consistency rate of 0.894 in complex sentences, demonstrating strong syntactic alignment capability. Syntax-Transformer performs well in simple sentences but shows a noticeable decline in performance in complex sentences. In Fig 5c, the proposed model has the lowest reconstruction cost in cross-linguistic sentences, at 0.214, significantly outperforming other models. Syntax-BERT and Syntax-GNN exhibit higher costs in complex sentences. Fig 5d reveals that the proposed model attains the best balance in complex sentences, with a score of 0.943, reflecting its strong ability to maintain the syntactic structure post-translation. Dual-Syntax-Encoder performs slightly worse in cross-linguistic sentences.

## Discussion

In the performance comparison experiments, the optimized BLSTM-CRF model demonstrates notable advantages in identifying syntactic complexity in both complex and cross-linguistic sentences. This result mainly stems from the model’s explicit integration of various syntactic features and targeted architectural improvements, which enhance its capacity to model key aspects such as syntactic depth, dependency relations, and embedded clause levels. Experimental results show that the model achieves strong performance across multiple indicators, including accuracy, precision, dependency coverage, and syntactic completeness. These outcomes indicate the model’s robust ability to analyze and construct complex syntactic structures. However, the optimized model does not outperform all other models across every sentence type or evaluation indicator. For instance, models such as Syntax-GNN and Syntax-Transformer exhibit faster convergence and higher accuracy when processing simple sentences, suggesting that they are more effective for texts with straightforward structures and stable word order. On certain datasets, these models even achieve performance comparable to or better than the optimized BLSTM-CRF. Moreover, the Dual-Syntax-Encoder shows an advantage in cross-linguistic syntactic alignment. It performs stably when mapping between source and target language structures. Nonetheless, it still faces challenges in syntactic tree reconstruction cost and structural balance, leading to slightly weaker overall performance. A comprehensive analysis of the eight indicators reveals that although the optimized BLSTM-CRF does not consistently lead in all dimensions and tasks, it maintains high stability and accuracy in handling complex and cross-linguistic syntactic structures. Compared to other models, it shows better syntactic adaptability and modeling consistency when facing deeply embedded structures and long-distance dependencies. Overall, the proposed model demonstrates a strong integrated advantage in syntactic modeling accuracy, structural preservation, and adjustment abilities. It is particularly well-suited for high-complexity translation scenarios and provides more robust support for translation tasks involving intricate syntax.

Compared to the research by Han and Lu (2023), this study places greater emphasis on the adaptability of cross-lingual syntactic features in the syntactic complexity recognition task [40]. Their research primarily focused on modeling syntactic complexity within a single language, using indicators such as dependency relations and syntactic tree depth to assess the complexity of different sentences. However, their study did not address the critical issue of cross-lingual syntactic transformation. In contrast, this study enhances the model’s performance in cross-lingual syntactic complexity recognition tasks by incorporating multilingual pre-trained word embeddings into the BLSTM-CRF structure. This method notably improves the model’s adaptability in areas such as syntactic structure adjustment and long-distance dependency transformation. Steigerwald et al. (2022) primarily combined traditional LSTM and CNN for syntactic complexity modeling. Although this method could capture sentence structure information to some extent, it had limited ability to handle long-distance dependencies and faced challenges in learning complex syntactic layers [41]. Compared to the study by Steigerwald et al. (2022), this study adopts a more comprehensive optimization strategy for modeling complex syntactic structures. By introducing attention mechanisms and global syntactic constraints, this study enhances the model’s ability to parse deep syntactic structures, maintain syntactic consistency, and recognize complex syntactic patterns. As a result, it demonstrates improved stability when handling long sentences and embedded clauses, with more accurate modeling of cross-lingual syntactic structure transformation.

In short, this study makes notable advancements in cross-lingual syntactic complexity recognition, long-distance dependency modeling, and deep syntactic structure parsing. Compared to previous research, this study strengthens the model’s ability to recognize syntactic complexity within a single language. Also, it enhances the model’s adaptability and generalization ability in translation tasks, offering new methods and ideas for future MT optimization research.

## Conclusion

This study addresses the problem of syntactic complexity recognition and analysis in Chinese-English translation and proposes a solution based on the optimized BLSTM-CRF model. The model’s performance and syntactic complexity modeling capabilities are comprehensively evaluated through multidimensional indicators. The optimized BLSTM-CRF model performs excellently on critical indicators, such as dependency coverage rate, syntactic depth, and the embedded clause levels, particularly in complex and cross-linguistic sentence scenarios, demonstrating strong syntactic complexity modeling ability. The experimental results also illustrate that the proposed model better preserves syntactic structure consistency in translation tasks, especially in syntactic tree reconstruction costs and syntactic alignment consistency, outperforming the comparative models. Moreover, through multidimensional experimental validation, the proposed model proves effective in recognizing syntactic complexity in Chinese-English translation and holds significant practical value for translating complex syntactic structures and quality evaluation. Although the optimized BLSTM-CRF model shows remarkable improvement in syntactic complexity recognition tasks, some limitations remain and warrant further research and improvement. First, this study does not provide a detailed analysis of the model’s performance at different levels of syntactic complexity. For instance, complex syntactic structures, such as varying depths of embedded clauses and long-distance dependencies, may impact the model’s processing capability in diverse ways. The current study has not systematically compared and quantitatively analyzed these factors. Therefore, future research should further refine the dataset and evaluate the model’s recognition ability at various syntactic complexity levels, revealing its adaptability across varying levels of complexity. Second, the optimized BLSTM-CRF enhances the model’s ability to model complex syntax through the introduction of attention mechanisms, multitask learning, and global syntactic constraints. However, there may still be bottlenecks in processing highly complex syntactic structures. For example, the model may struggle to effectively learn the global structure of deeply embedded clauses or long-distance dependencies that span multiple phrases. As a result, it leads to instability in the annotation results or structural shifts during translation. However, the current study does not delve into the specific performance differences of the optimized BLSTM-CRF under various types of syntactic complexity. It also does not identify situations where the model’s recognition ability may decline. Hence, future research should explore the model’s limitations and identify key syntactic features that impact its performance. It should also attempt to incorporate more advanced syntactic encoding methods, such as GNN-based syntactic parsing, to enhance the model’s generalization ability. Additionally, this study primarily focuses on syntactic complexity recognition tasks without deeply discussing how different types of syntactic complexity affect translation accuracy. Syntactic complexity not only influences the understanding and processing within the translation system but may also pose challenges to syntactic reconstruction in the target language. Different syntactic complexity types, such as deeply embedded structures, long-distance dependencies, and imbalanced syntactic trees, may lead to issues in word order adjustment, information retention, and syntactic consistency within the translation model. However, the experimental design in this study does not specifically analyze the translation quality under different syntactic complexity conditions. Future research could further combine BLEU scores, syntactic error rates, and other indicators to assess the specific impact of syntactic complexity on translation quality. Moreover, it can explore different syntactic complexity control mechanisms, such as syntactic reconstruction or preprocessing methods before translation, to mitigate the negative impact of high syntactic complexity on translation outcomes.

In conclusion, this study optimizes syntactic complexity recognition but still faces some limitations. Future research should further analyze the model’s performance under varying levels of syntactic complexity and explore the challenges the optimized BLSTM-CRF faces in handling certain complexities. It should also clarify how different levels of syntactic complexity influence translation accuracy. These improvements help further refine the model’s adaptability and increase its value in MT tasks.

## Supporting information

S1 File Data (18).zip. (ZIP)
