# Supplementary material for: Syntactic complexity recognition and analysis in Chinese-English machine translation: A comparative study based on the BLSTM-CRF model
Source: PLoS One. 2025 Jun 12;20(6):e0325721. doi: 10.1371/journal.pone.0325721 (PMC12161555; doi:10.1371/journal.pone.0325721)
Supplement: S1 File — (ZIP) [file pone.0325721.s001.zip › ╩2╛▌░n/Code Description.docx]

1. Import the necessary libraries

- **Function:** Python libraries related to model construction, optimization, data processing, and evaluation are imported.
- **torch:** They are used for building, training, and optimizing deep learning models.
- **spacy:** Preprocessing of syntactic features is provided, such as word segmentation, part-of-speech (POS) tagging, and dependency extraction.
- **tqdm:** They are employed to display a progress bar during the training process for easy observation of training progress.

**2.** Setting up computing devices

- **Function:** These devices can automatically detect whether there is a Graphic Processing Unit (GPU), use GPU acceleration if available, otherwise use Center Processing Unit (CPU).
- **Advantage:** The computational efficiency is improved, especially when dealing with large-scale data.

3. Load the SpaCy model

 **Function:** The pre-trained language model of SpaCy is loaded for tokenization, POS tagging, and dependency extraction.

 **"en_core_web_sm"**: A small English corpus containing rich syntactic information.

4. Text preprocessing function

 **Function:** The input text is parsed into words, POS tagging, and dependencies.

 **Step-by-step analysis:**

- SpaCy's nlp is used to parse text.
- The text content, part-of-speech tagging (POS), and dependency relationships (Dependency) of each word are extracted.

 **Return:** The parsed tokens (word list), POS tag list, and dependency relationship list.

5. Feature coding function

 **Function:** Non-numerical features such as POS tagging and dependency relationships are converted into numerical codes for neural network processing.

 **Detail:**

- Features are encoded into integer values based on a specific vocabulary (VOCAB).
- "UNK" is used as the default encoding for unknown features.

6. Dataset definition

 **Function:** The dataset class is defined for loading and managing input text and its features.

 **Constructor function:**

- texts: Original input text.
- labels: Corresponding labels (such as POS tags or dependency relationships).
- pos_vocab and dep_vocab: Vocabulary used for feature encoding.

 **Detail:**

- __len__: Return the size of the dataset.
- __getitem__: Preprocess the data of the specified index and return tokens, POS encoding, dependency encoding, and labels.

7. Definition of the BLSTM-CRF model

 **Function:** The optimized BLSTM-CRF model is defined, including multi feature input and sequence annotation functions.

 **Module composition:**

- **Embedding layer:** Words, POS tags, and dependency relationships are embedded separately to map discrete features to a vector space.
- **LSTM layer:** Bidirectional LSTM is used to capture contextual information of the input sequence.
- **Conditional random field (CRF) layer:** CRF is utilized to achieve global sequence optimization, ensuring syntactic consistency of annotated results.

 **Model parameter:**

- vocab_size, pos_size, dep_size: The size of the vocabulary list for the three characteristics.
- embedding_dim: The dimension of the embedding vector.
- hidden_dim: The dimension of the LSTM hidden layer.
- tagset_size: Output the size of the tag set.

8. CRF loss function

- **Function:** The CRF loss is calculated and the global score of sequence annotation is optimized.
- **Detail:**
  - compute_seq_score: The score for the given label sequence is calculated.
  - compute_log_partition: The log-partition function is calculated for normalization.

9. Model training function

- **Function:** The training process of the model is defined, including forward propagation, loss calculation, backpropagation, and parameter updates.
- **Detail:**
  - The train_rader is traversed to obtain training data.
  - Optimizers such as Adam are used to update model parameters.
  - The average loss value for each epoch is printed.

10. Model evaluation function

 **Function:** The performance, computational accuracy, and precision of the model on the validation or test set are evaluated.

 **Detail:**

- Each batch of data is predicted.
- The predicted and actual values are statistically analyzed to calculate the overall performance indicators.

11. Main program

 **Function:** The running process is set up, including data loading, model initialization, training, and evaluation.

 **Process:**

1. A TranslationDataset is constructed using forged data.
2. The model (BLSTMCRF) and optimizer are initialized.
3. train_madel is called for model training.
4. evaluate_model is called for model evaluation.
