# Supplementary material for: Syntactic complexity recognition and analysis in Chinese-English machine translation: A comparative study based on the BLSTM-CRF model
Source: PLoS One. 2025 Jun 12;20(6):e0325721. doi: 10.1371/journal.pone.0325721 (PMC12161555; doi:10.1371/journal.pone.0325721)
Supplement: S1 File — (ZIP) [file pone.0325721.s001.zip › ╩2╛▌░n/Dataset Description.docx]

**Workshop on Machine Translation (WMT) Chinese-English Parallel Corpus**

**1. Dataset introduction**
The Workshop on Machine Translation (WMT) Chinese-English Parallel Corpus is a high-quality bilingual dataset provided by the World Translation Conference. It is widely used for training, evaluation, and research in machine translation models and related natural language processing tasks. The dataset includes Chinese-English bilingual sentence pairs from various domains, covering everyday language, news reports, academic papers, legal documents, and more, making it highly representative and generalizable.

**2. Data source and composition**
The WMT dataset combines high-quality parallel corpora from multiple sources, ensuring data diversity and coverage. The main sources include:

- **ParaCrawl:**
  The parallel corpus of Chinese and English crawled from the internet is filtered and cleaned to ensure the alignment quality of sentence pairs.
- **News Commentary:**
  A Chinese English translated text containing news commentary, covering professional terminology and news language in news reporting.
- **UN Parallel Corpus:**
  The Chinese English translation corpus from United Nations documents has a high degree of professionalism and linguistic standardization.
- **Wiki Titles:**
  It contains Wikipedia title translation data, covering a wide range of fields but with a relatively simple language style.
- **WikiMatrix:**
  A large-scale collection of parallel sentence pairs, generated based on Wikipedia data, is suitable for training deep learning models.
- **CCMT:**
  The bilingual corpus provided by the Chinese Information Processing Conference (CCMT) is biased towards technical documents and scientific research fields.

**3. Data size and format**

- **Data size:**
  - Total number of sentence pairs: approximately 25 million bilingual sentence pairs in Chinese and English.
  - Data volume: The total size of the original text file is about tens of GB, and the specific size varies depending on the sub-corpus used and the degree of cleaning.
- **Data format:**
  The data is stored in simple text format (TXT) or TSV/CSV format, with each line representing a pair of Chinese English sentence pairs. The representation is usually as follows:
  - The left side is a Chinese sentence; the right side is the corresponding English sentence, separated by a tab or separator in the middle.
  - Data example:

Copy code

Chinese sentence 1 English sentence 1

Chinese sentence 2 English sentence2

- **Tokenization and annotation:**
  - The raw data has not undergone tokenization processing, and users need to tokenize Chinese data according to task requirements.
  - English data is usually annotated according to standard English word tokenization methods.

**4. Characteristics of the dataset**

1. **Diversity:**
   The dataset covers language data from multiple fields, including news, law, academia, technology, etc., and can support machine translation research in multiple scenarios.
2. **High quality:**
   The data is processed through a combination of automatic alignment and manual review, ensuring the accuracy and semantic consistency of sentence translation.
3. **Differences in language structure:**
   This dataset contains typical features of both Chinese and English languages:
   - **Chinese:** It is mostly a semantic cohesion with a shallow syntactic level, relying on context to express semantics.
   - **English:** It is mostly a formal cohesion with deep syntactic levels, relying on grammatical rules to express semantics.
4. **Cross-disciplinary representativeness:**
   The data comes from multiple industries and fields, covering a wide range of commonly used vocabulary and proprietary terms, offering a foundation for the model's generalization ability.
5. **Large scale:**
   The data volume of 25 million sentence pairs provides a rich data foundation for training deep learning models, especially suitable for pre-training and fine-tuning.

**5. Data usage and processing**

1. **Download and obtain:**
   The dataset can be downloaded through the official WMT platform or cooperative resource providers, and some sub-corpora can also be obtained through third-party links.
   Download link: <https://www.mdpi.com/>
2. **Data pre-processing:**
   - **Cleaning:** Duplicate and incomplete sentence pairs, or semantically mismatched sentences are removed.
   - **Tokenization:** Chinese data is tokenized and standardized with English annotations.
   - **Alignment:** It is necessary to check whether the sentence pairs are aligned correctly and delete sentences with translation errors.
3. **Division:**
   - The dataset is usually divided into training, validation, and testing sets, with a common division ratio of 80:10:10.
4. **Applicable scenarios:**
   - Training and testing of machine translation models such as Transformer and BLSTM-CRF.
   - Experimental data support for cross-linguistic tasks such as syntactic complexity analysis and syntactic alignment research.

**6. The value of datasets in the study of syntactic complexity**

1. **Comparison of syntactic structures:**
   The dataset contains bilingual sentence pairs in Chinese and English with significant syntactic differences, which helps to study the conversion patterns of syntactic structures between the two languages.
2. **Analysis of complex syntactic features:**
   The dataset contains a large number of long sentences, nested clauses, and sentences with complex dependency relations, providing real data support for the recognition and analysis of syntactic complexity.
3. **Translation consistency evaluation:**
   The dataset can be used to analyze the shortcomings of machine translation systems in handling syntactic complexity and help improve the model's syntactic alignment ability.
4. **Multi-dimensional analysis support:**
   The dataset has diverse fields and covers various syntactic features, making it suitable for research from multiple dimensions such as dependency relations, nested levels, and branching structures.

**7. License and copyright**

- The WMT dataset is an open resource, but its use must comply with the relevant regulations of the data provider to ensure legality and compliance. Especially in research involving syntactic complexity recognition and analysis, the data sources used must be cited and the copyright of the sub-corpus must be appropriately labeled. For example, as an important data source in the field of Chinese information processing, the CCMT corpus needs to indicate its source when analyzing its dependency relations and syntactic complexity to ensure the traceability and academic standardization of research results.
- This study strictly followed the requirements of the data provider in the experiment and standardized the management of corpus usage and result publication. It provides sustainable legal and ethical guarantees for the research on syntactic complexity recognition in Chinese English translation.

**Conclusion**
The WMT Chinese-English Parallel Corpus provides a solid data foundation for machine translation and natural language processing research due to its large scale, high quality, and diversity. In the research of syntactic complexity recognition and analysis, this dataset is particularly suitable as experimental basic data, which can support various research needs from capturing syntactic features to optimizing translation quality.
