# Supplementary figures and images for: Syntactic complexity recognition and analysis in Chinese-English machine translation: A comparative study based on the BLSTM-CRF model
Source: PLoS One. 2025 Jun 12;20(6):e0325721. doi: 10.1371/journal.pone.0325721 (PMC12161555; doi:10.1371/journal.pone.0325721)

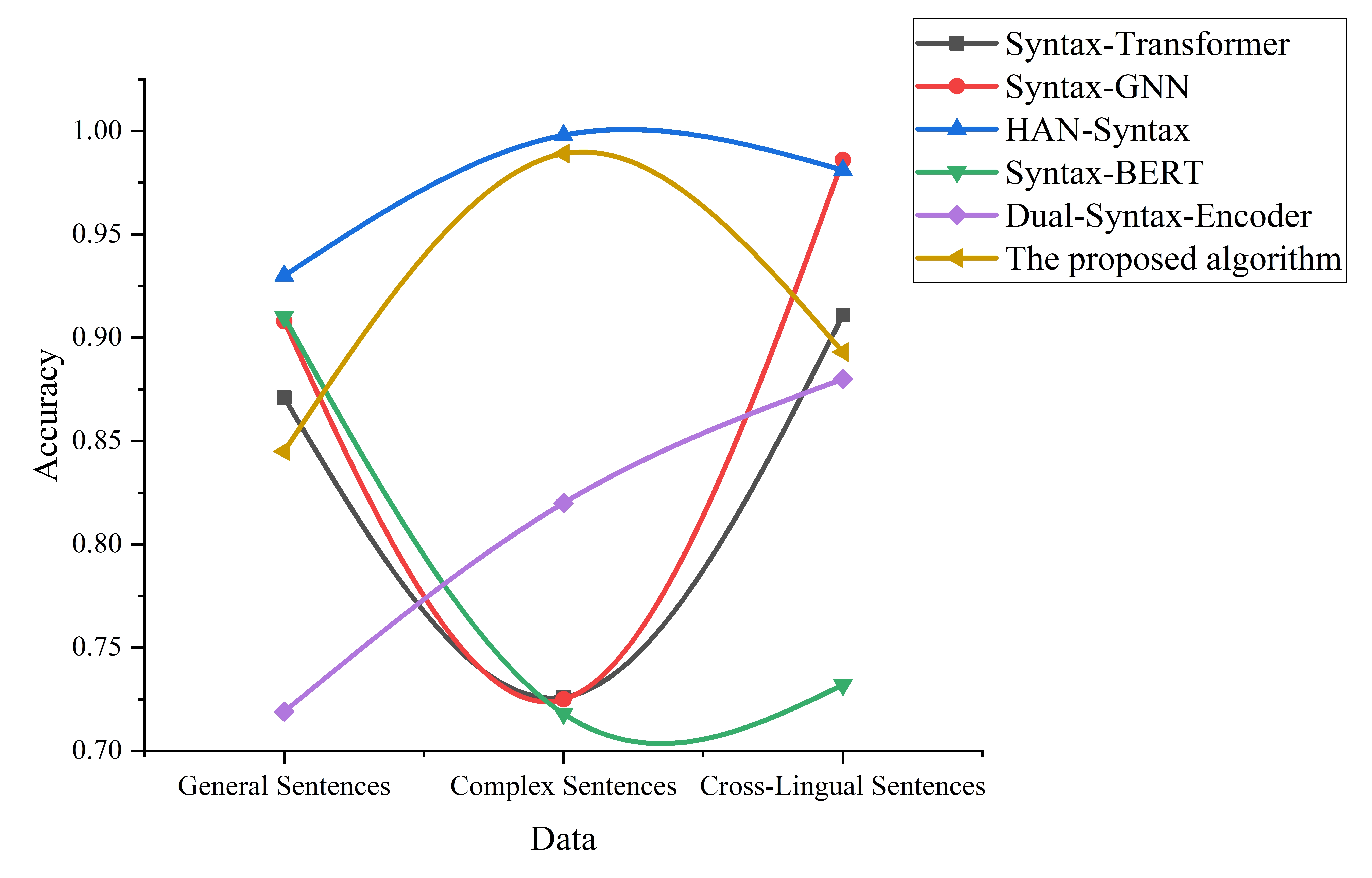

Supplement: S1 File — (ZIP) [file pone.0325721.s001.zip › ╩2╛▌░n/Figure2a.jpg]

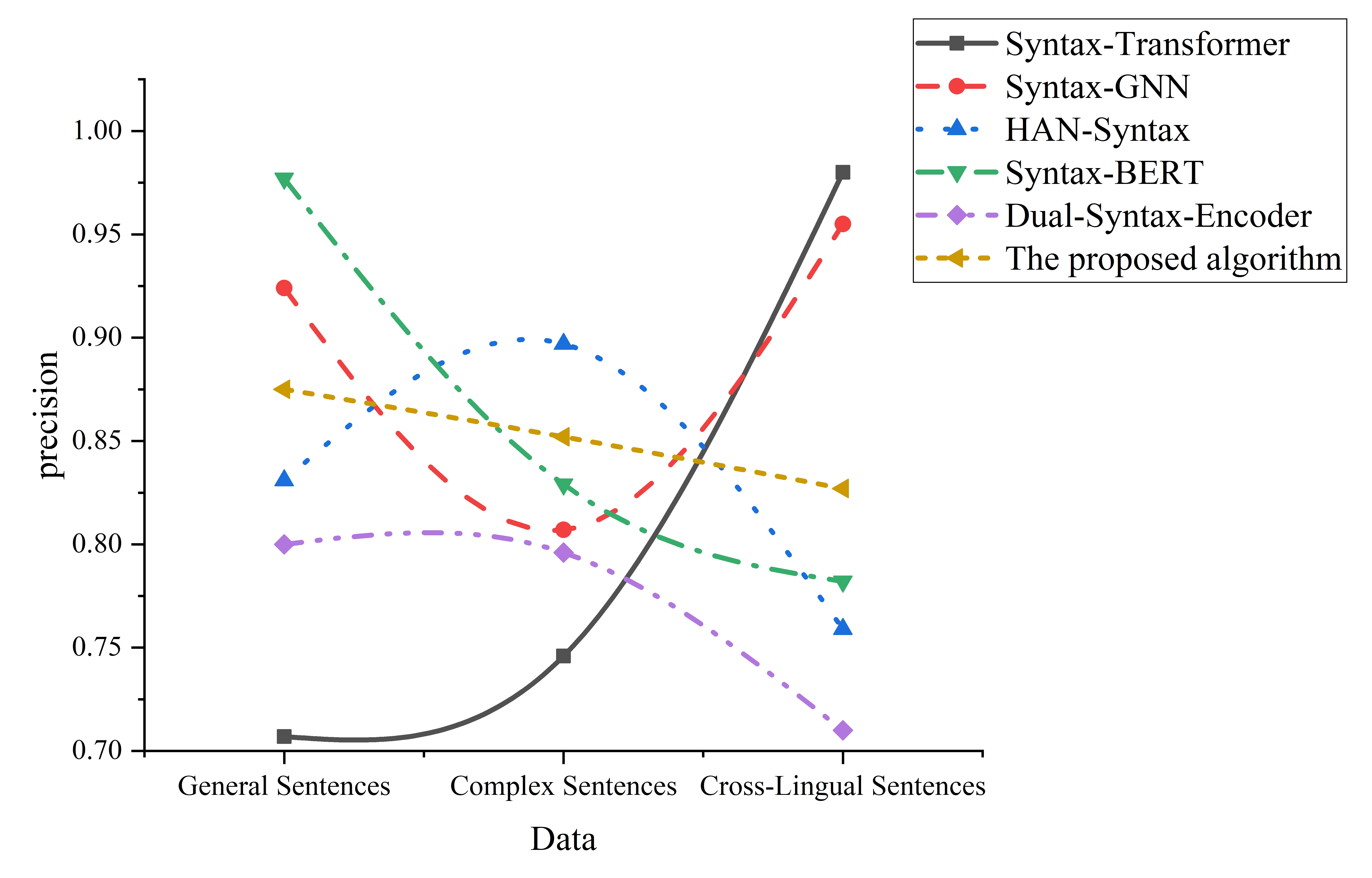

Supplement: S1 File — (ZIP) [file pone.0325721.s001.zip › ╩2╛▌░n/Figure2b.jpg]

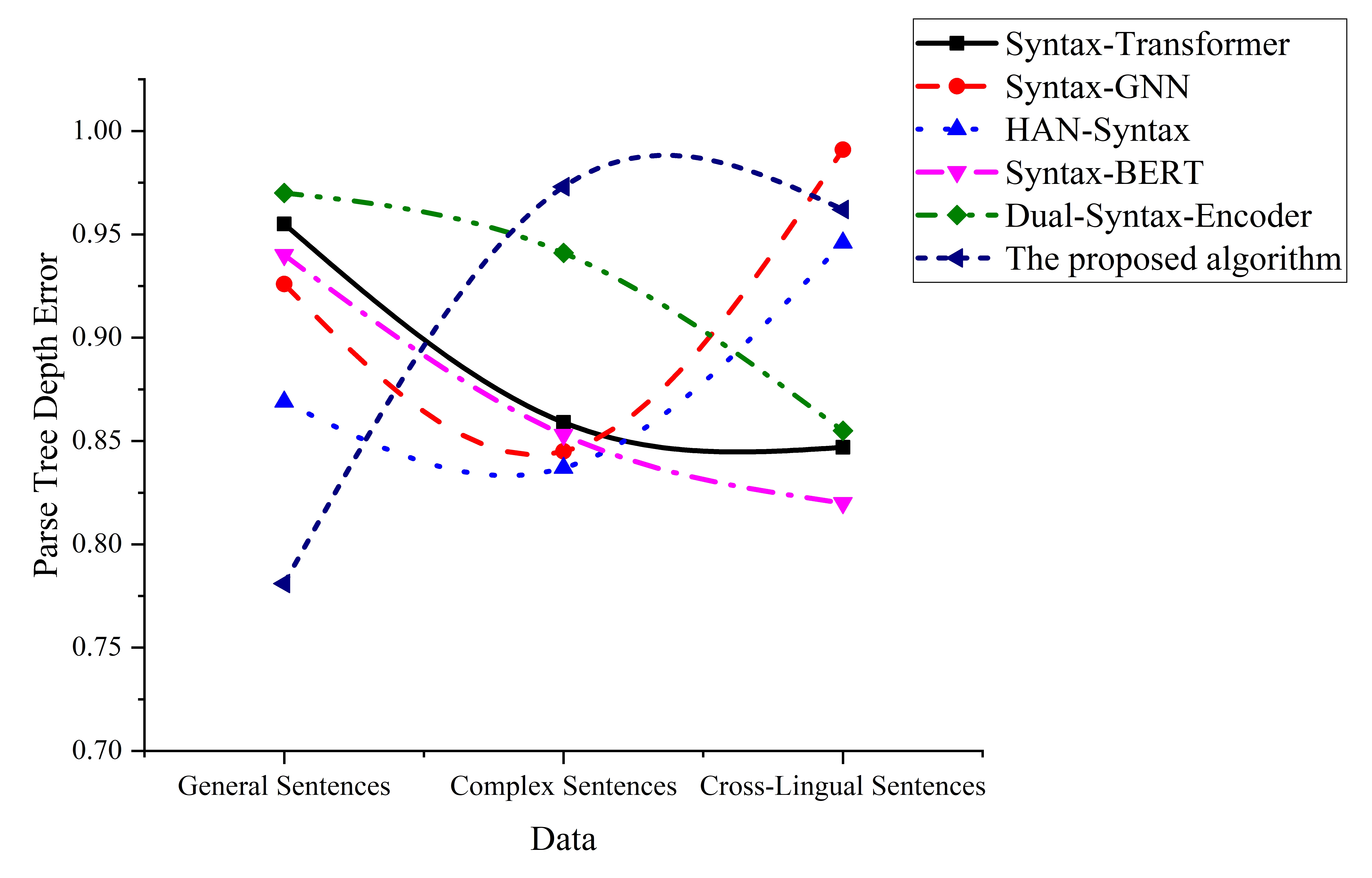

Supplement: S1 File — (ZIP) [file pone.0325721.s001.zip › ╩2╛▌░n/Figure2c.jpg]

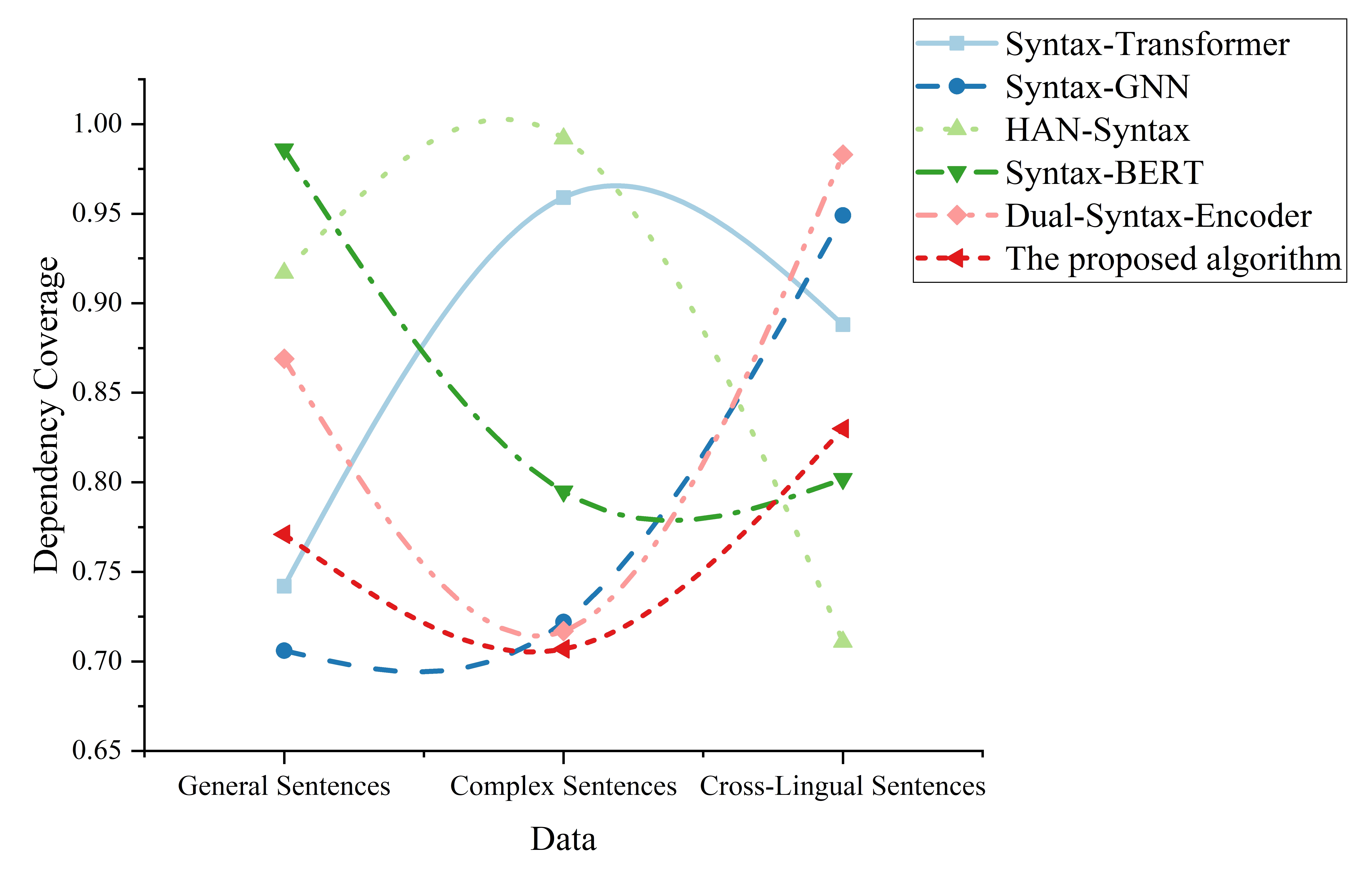

Supplement: S1 File — (ZIP) [file pone.0325721.s001.zip › ╩2╛▌░n/Figure2d.jpg]

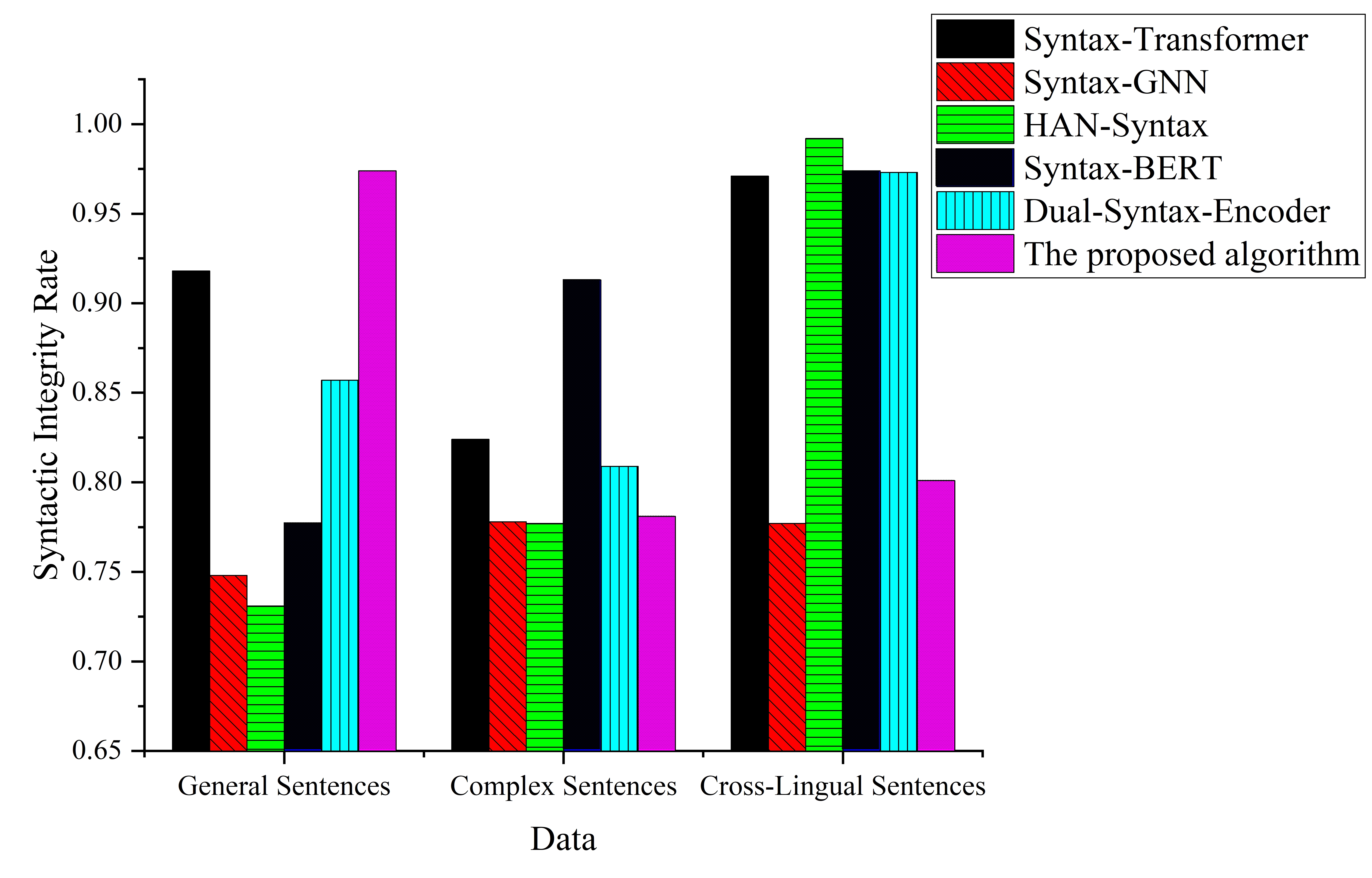

Supplement: S1 File — (ZIP) [file pone.0325721.s001.zip › ╩2╛▌░n/Figure3a.jpg]

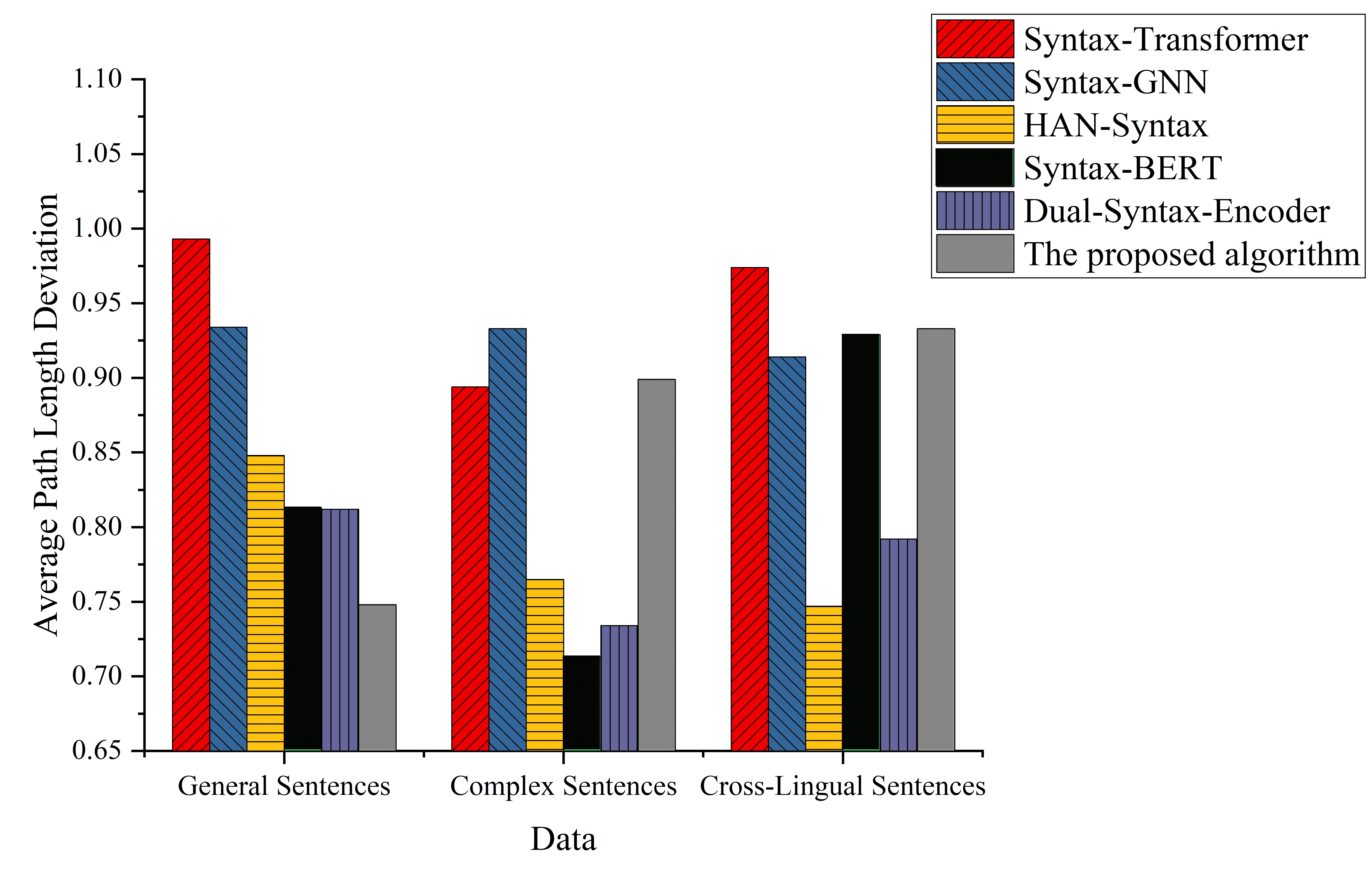

Supplement: S1 File — (ZIP) [file pone.0325721.s001.zip › ╩2╛▌░n/Figure3b.jpg]

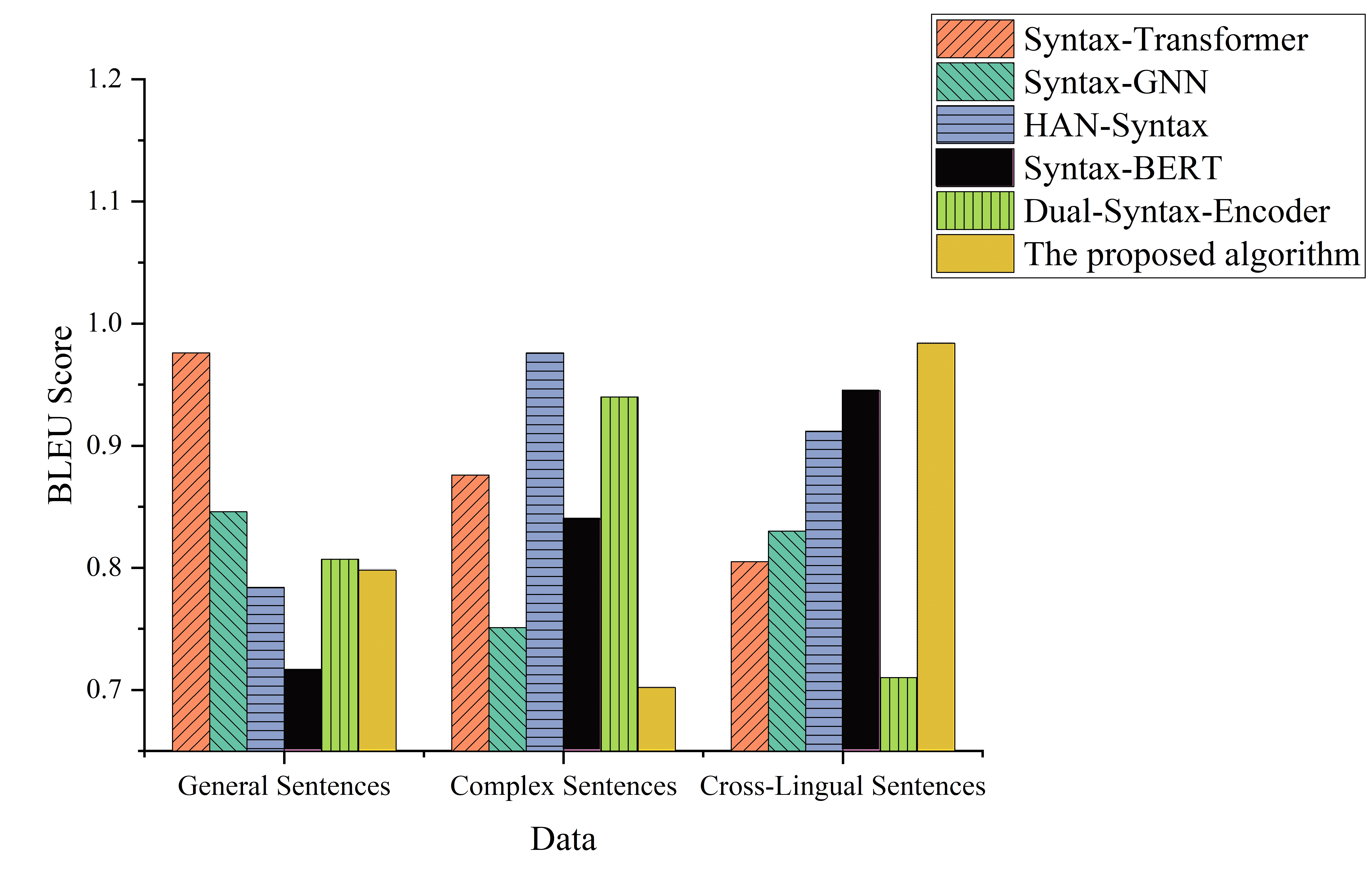

Supplement: S1 File — (ZIP) [file pone.0325721.s001.zip › ╩2╛▌░n/Figure3c.jpg]

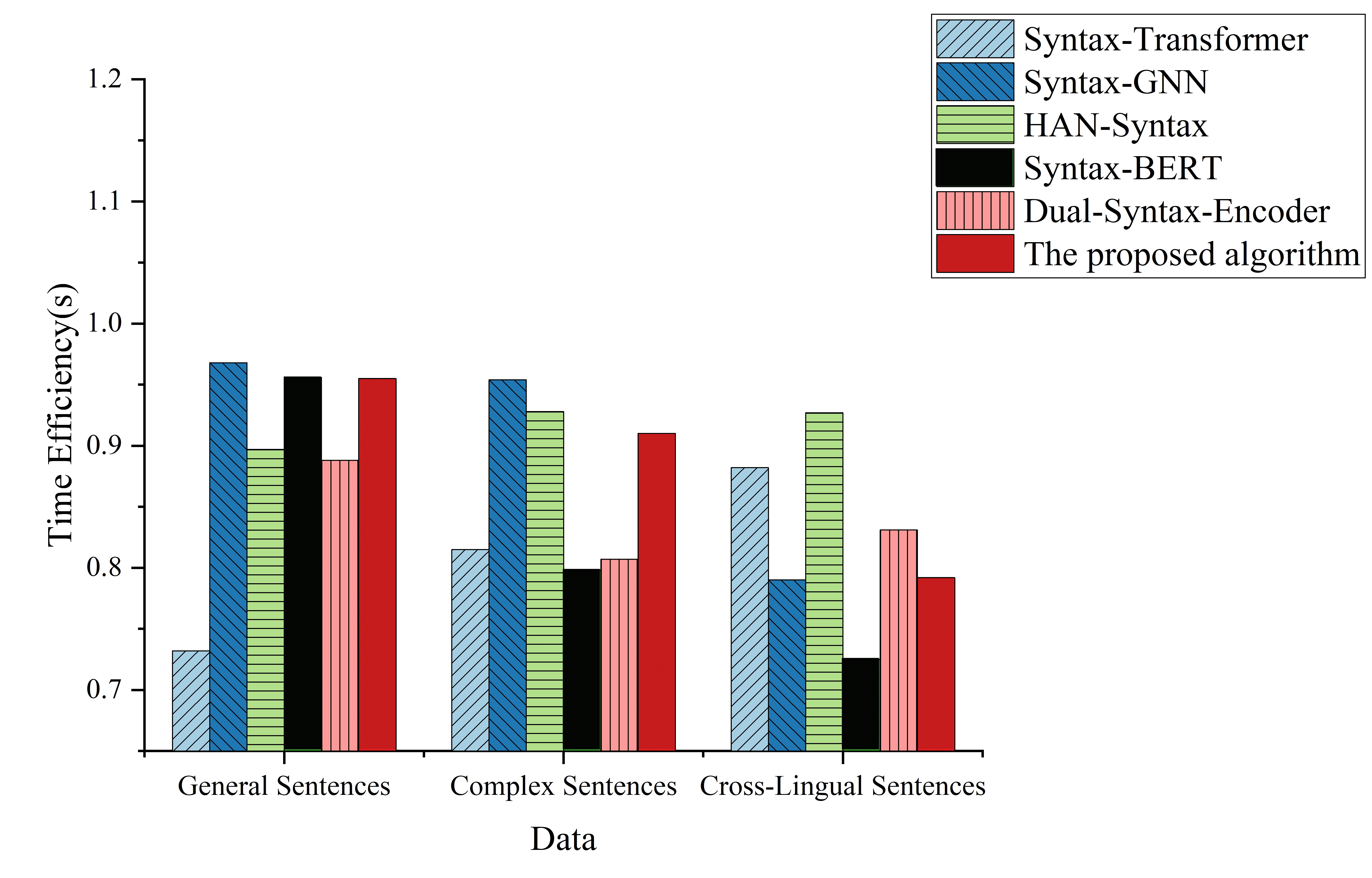

Supplement: S1 File — (ZIP) [file pone.0325721.s001.zip › ╩2╛▌░n/Figure3d.jpg]

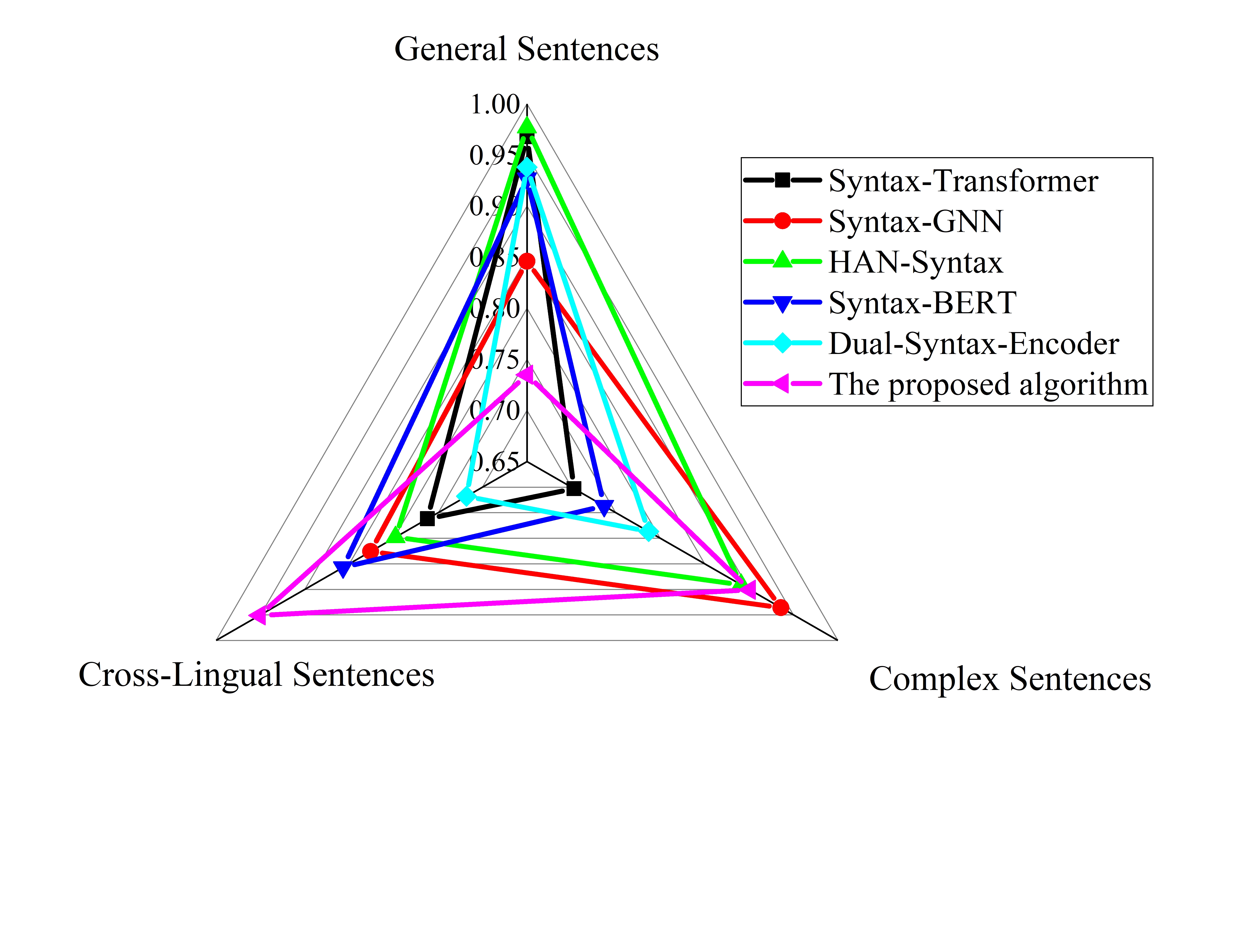

Supplement: S1 File — (ZIP) [file pone.0325721.s001.zip › ╩2╛▌░n/Figure4a.jpg]

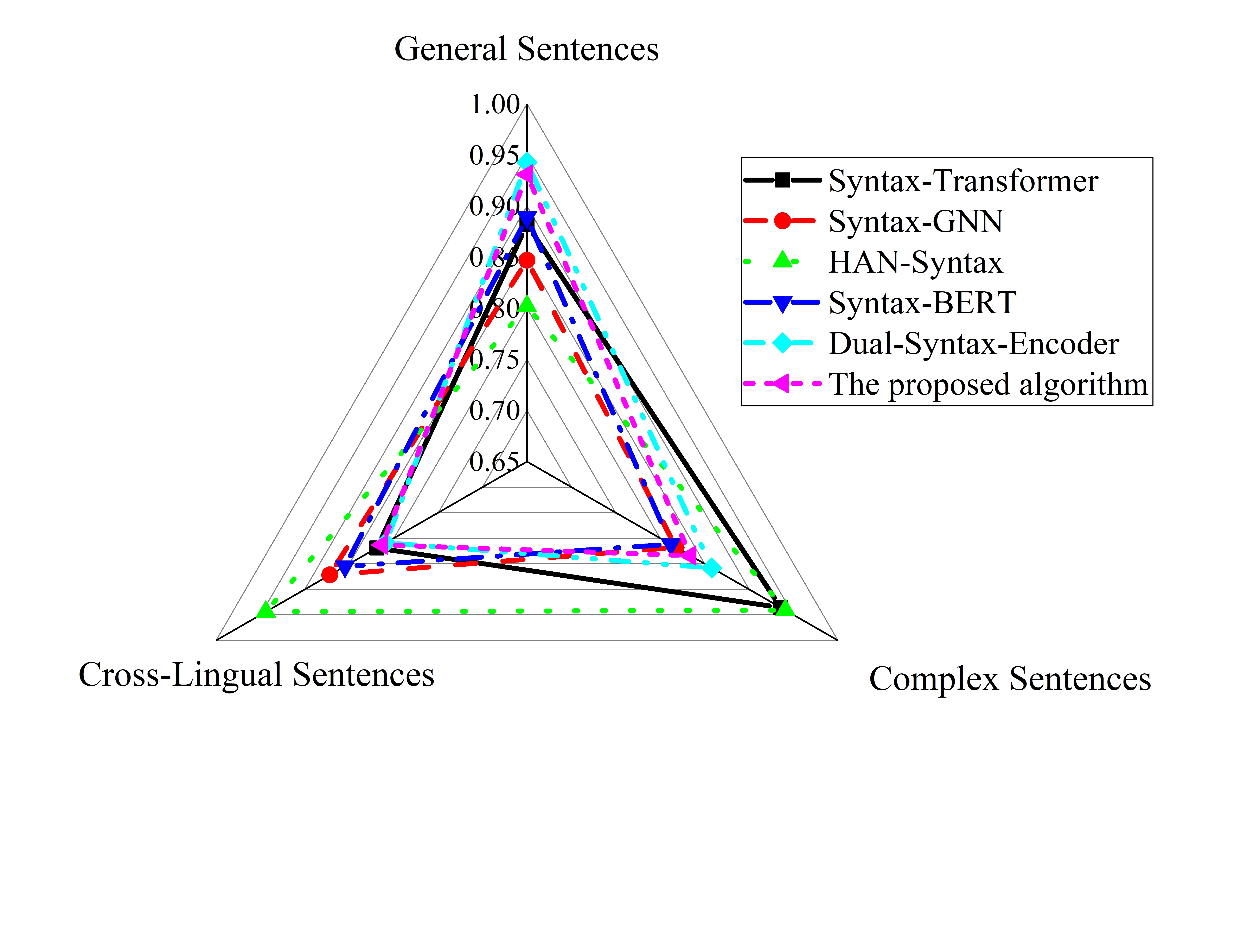

Supplement: S1 File — (ZIP) [file pone.0325721.s001.zip › ╩2╛▌░n/Figure4b.jpg]

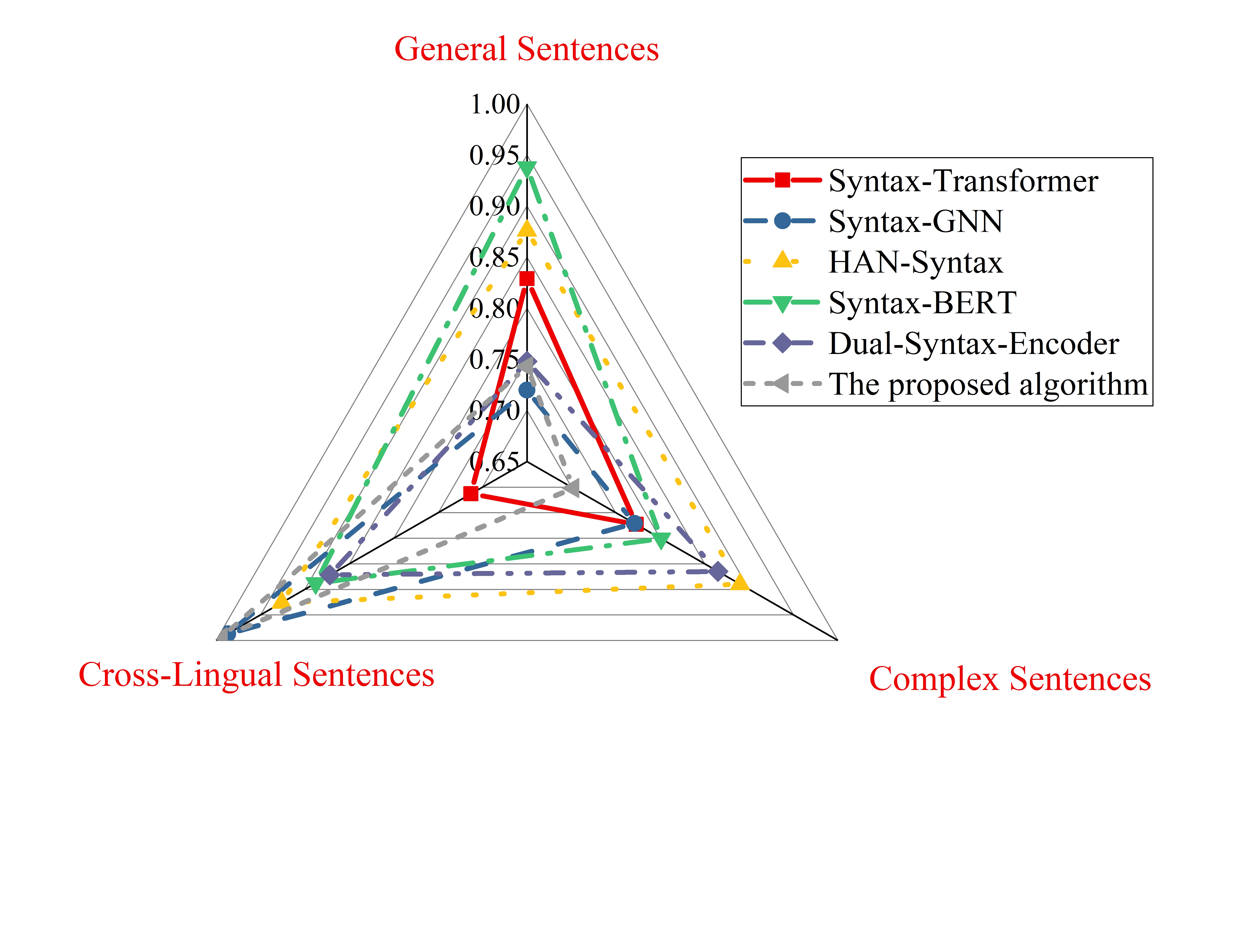

Supplement: S1 File — (ZIP) [file pone.0325721.s001.zip › ╩2╛▌░n/Figure4c.jpg]

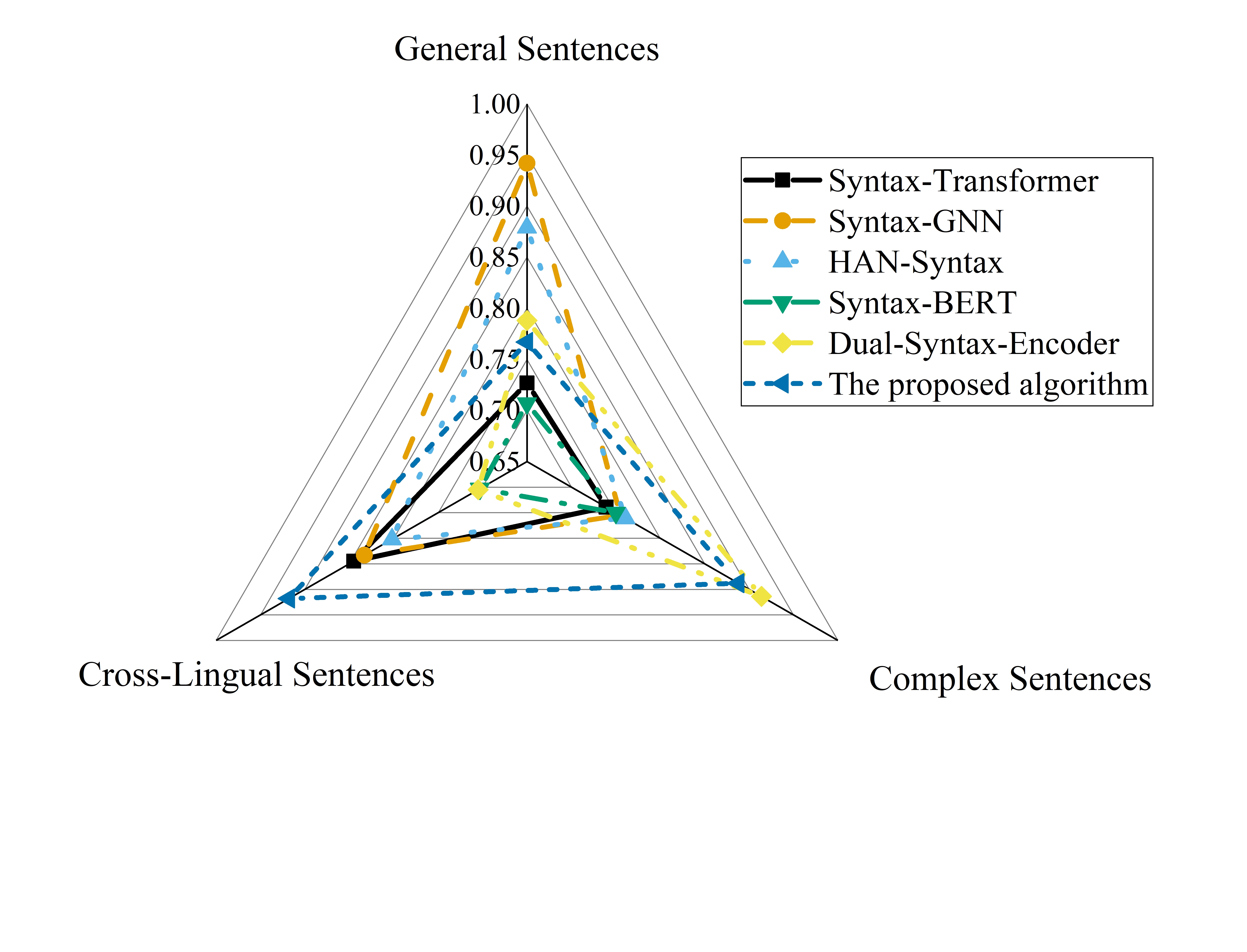

Supplement: S1 File — (ZIP) [file pone.0325721.s001.zip › ╩2╛▌░n/Figure4d.jpg]

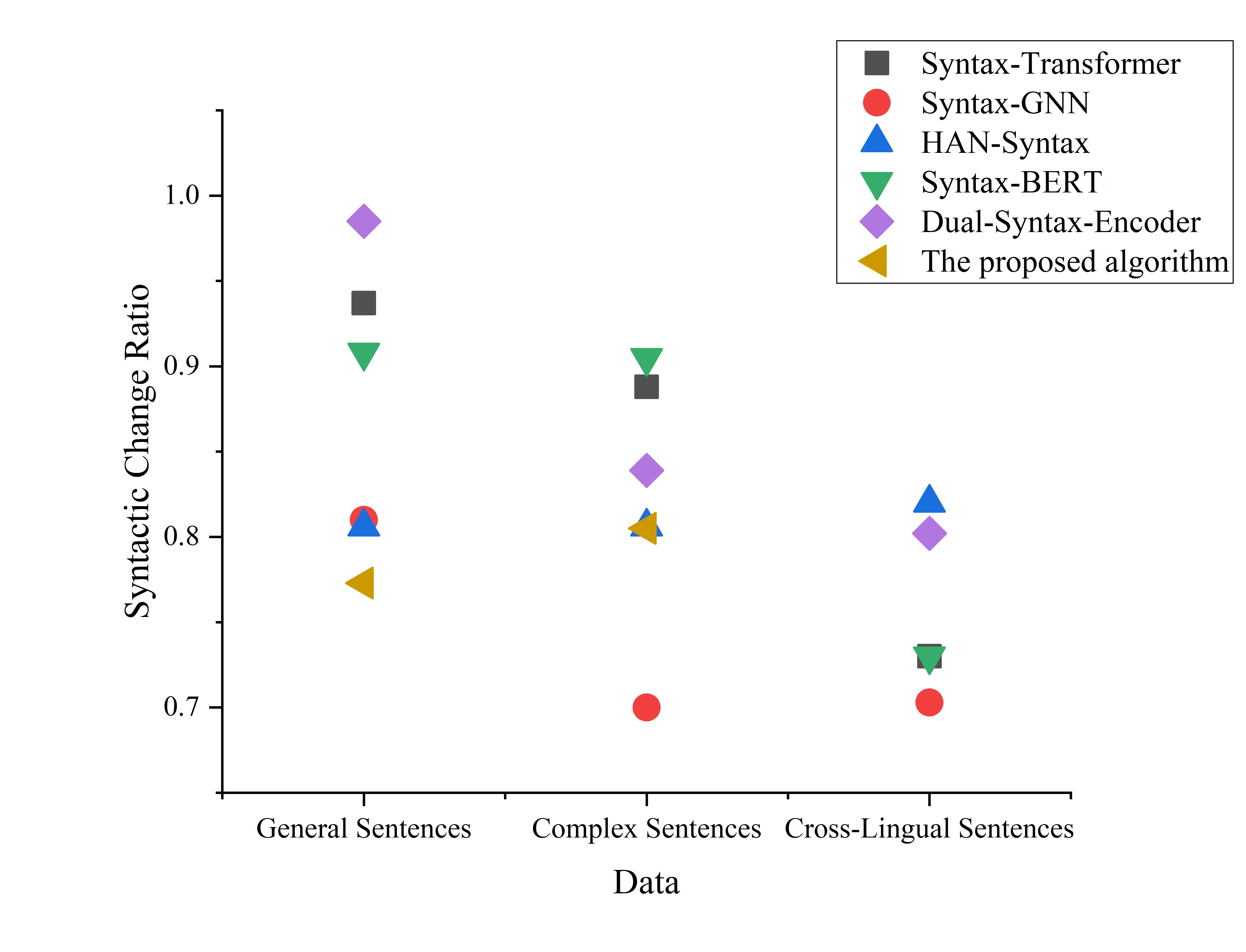

Supplement: S1 File — (ZIP) [file pone.0325721.s001.zip › ╩2╛▌░n/Figure5a.jpg]

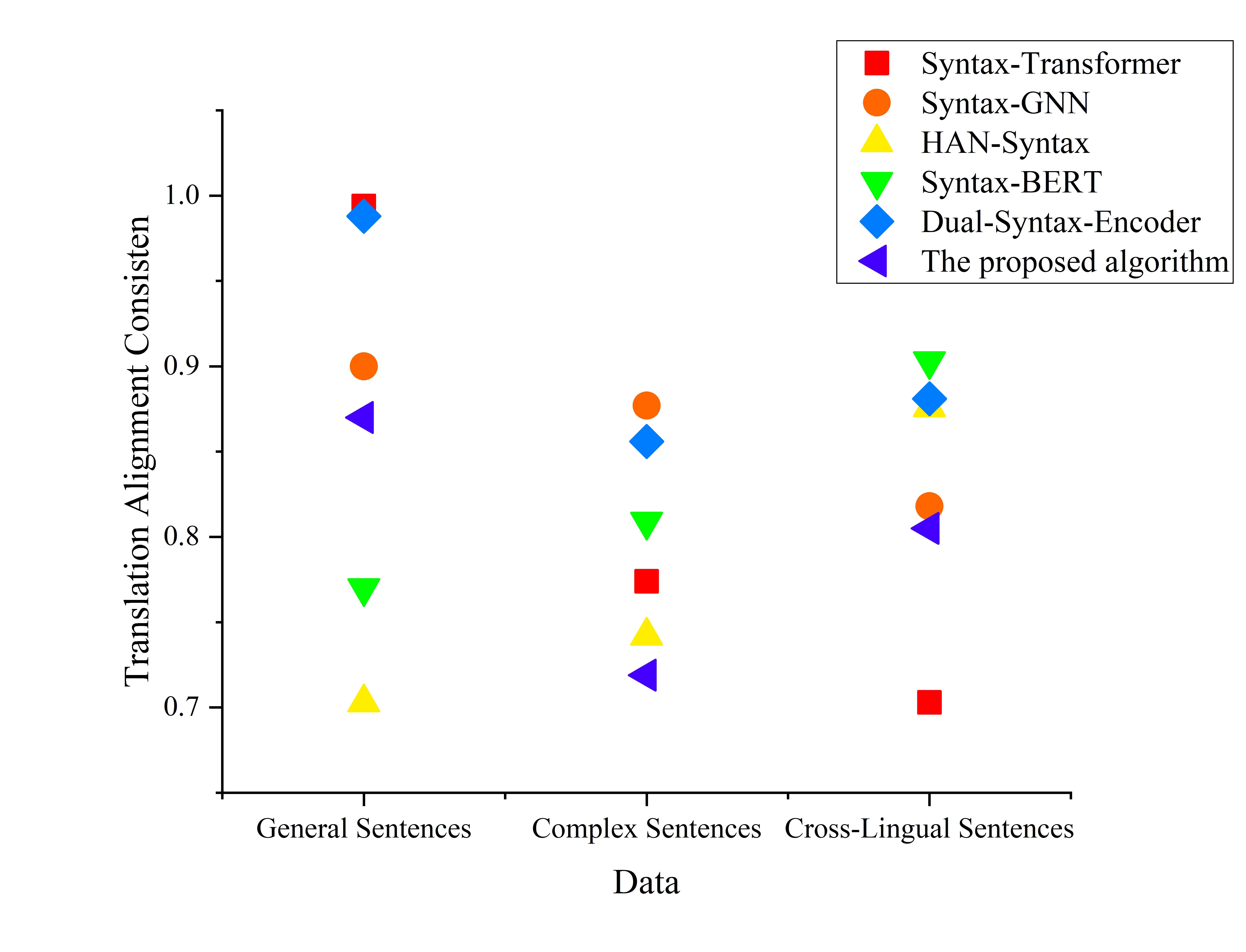

Supplement: S1 File — (ZIP) [file pone.0325721.s001.zip › ╩2╛▌░n/Figure5b.jpg]

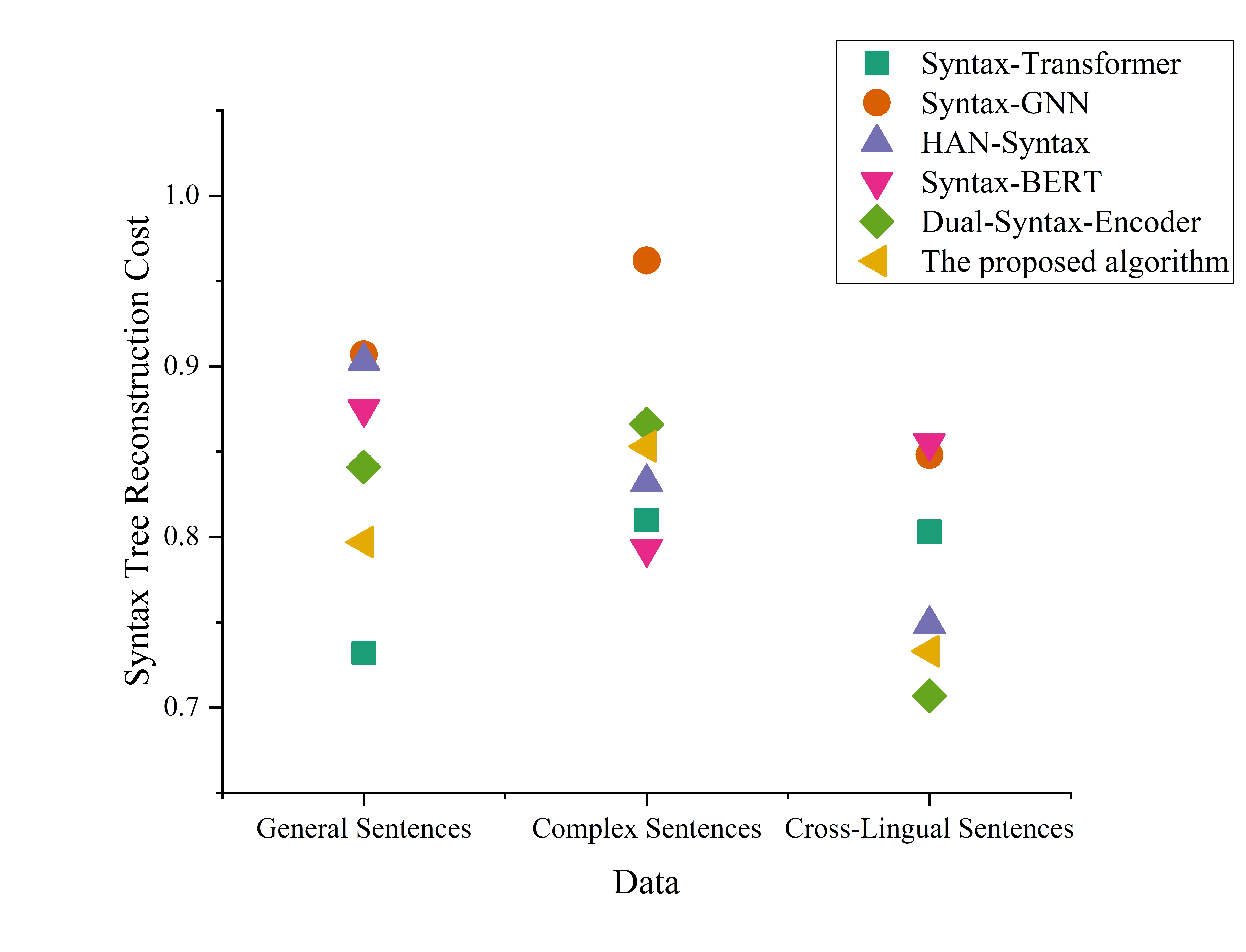

Supplement: S1 File — (ZIP) [file pone.0325721.s001.zip › ╩2╛▌░n/Figure5c.jpg]

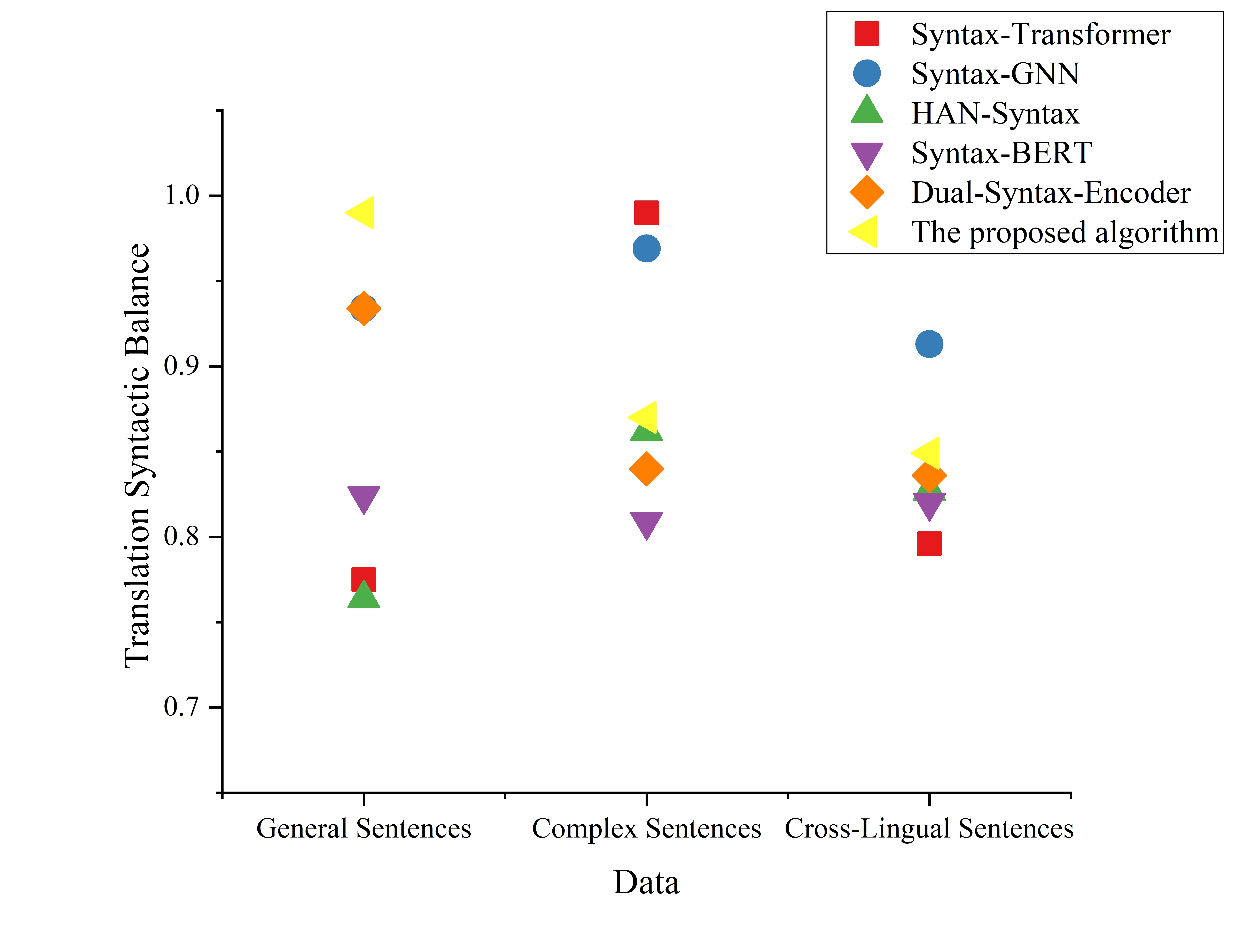

Supplement: S1 File — (ZIP) [file pone.0325721.s001.zip › ╩2╛▌░n/Figure5d.jpg]
